# Identification of new cold tolerant Zoysia grass species using high-resolution RGB and multi-spectral imaging

**DOI:** 10.1038/s41598-023-40128-2

**Published:** 2023-08-14

**Authors:** Ki-Bon Ku, Sheikh Mansoor, Gyung Deok Han, Yong Suk Chung, Thai Thanh Tuan

**Affiliations:** 1 Department of Plant Resources and Environment, Jeju National University, Jeju, 63243 Republic of Korea; 2 Department of Practical Arts Education, Cheongju National University of Education, Cheongju, 28708 Republic of Korea

**Keywords:** Plant sciences, Climate sciences, Environmental sciences

## Abstract

Zoysia grass (*Zoysia spp*.) is the most widely used warm-season turf grass in Korea due to its durability and resistance to environmental stresses. To develop new longer-period greenness cultivars, it is essential to screen germplasm which maintains the greenness at a lower temperature. Conventional methods are time-consuming, laborious, and subjective. Therefore, in this study, we demonstrate an objective and efficient method to screen maintaining longer greenness germplasm using RGB and multispectral images. From August to December, time-series data were acquired and we calculated green cover percentage (GCP), Normalized Difference Vegetation Index (NDVI), Normalized Difference Red Edge Index (NDRE), Soil-adjusted Vegetation Index (SAVI), and Enhanced Vegetation Index (EVI) values of germplasm from RGB and multispectral images by applying vegetation indexs. The result showed significant differences in GCP, NDVI, NDRE, SAVI, and EVI among germplasm (*p* < 0.05). The GCP, which evaluated the quantity of greenness by counting pixels of the green area from RGB images, exhibited maintenance of greenness over 90% for August and September but, sharply decrease from October. The study found significant differences in GCP and NDVI among germplasm. san208 exhibiting over 90% GCP and high NDVI values during 153 days. In addition, we also conducted assessments using various vegetation indexes, namely NDRE, SAVI, and EVI. san208 exhibited NDRE levels exceeding 3% throughout this period. As for SAVI, it initially started at approximately 38% and gradually decreased to around 4% over the course of these days. Furthermore, for the month of August, it recorded approximately 6%, but experienced a decline from about 9% to 1% between September and October. The complementary use of both indicators could be an efficient method for objectively assessing the greenness of turf both quantitatively and qualitatively.

## Introduction

Zoysia grass, belonging to the Zoysia genus, is a popular turf grass used in Korea and consists of about 16 species. It is widely distributed along the Pacific from the northeast Asian region, such as Korea and Japan, showing a temperate climate, to the Southeast Asian region, such as the Philippines and Thailand, showing a tropical climate^1^. It grows low due to its strong lateral and underground spread and blooms in May to June and fruits in June to July but mostly relies on vegetative propagation because of its low germination rate. Zoysia grass in Korea maintains its green color for about six months from mid-April to mid-October, but during winter, it goes dormant and turns yellow or brown^2^. It has strong resistance to biological and non-biological stressors such as disease, insects, drought, and wear, making it easy to manage. However, it has slower growth, slower recovery rate after damage, and delayed green-up in the spring, as disadvantages^3^. The Zoysia grass species that grow natively in Korea include *Zoysia japonica, Z. matrella, Z. sinica, Z. macrostachya*, and hybrid Zoysia grass. Among these, *Z. japonica*, known as Korean lawn grass, is the most widely used species in Korea due to its strong adaptability to the environment, despite its slightly rough texture and low-density formation. *Z. matrella* is also commonly used in Korea, and it has received high evaluations in terms of turf quality due to its soft texture caused by fine leaves and excellent sod-forming ability. *Z. sinica* and *Z. macrostachya* are not commercially used yet, but their strong salt tolerance makes them valuable breeding materials. Hybrid Zoysia grass, showing intermediate characteristics between *Z. japonica* and *Z. matrella* or *Z. sinica*, has been confirmed to grow in the southern region of Korea. *Z. tenuifloria* is being used in Korea, although its native growing area has not been confirmed^2,4^.

The surrounding environment of grass has a significant impact on its growth, including factors such as moisture, temperature, light, and soil. When these environmental factors are not suitable for grass growth, such as in cases of drought, heat, cold, shade, or high salt, the grass becomes stressed, which can inhibit its growth and reduce its quality^5–7^. Therefore, many researchers have studied the physiological, morphological, and metabolic responses of grass to various environmental stresses to improve its internal adaptability^8^. Studies have been conducted to measure various parameters using the altered responses to stress as an indicator to identify grass species with excellent internal adaptability. The phenotypic diversity of perennial ryegrass (*Lolium perenne* L.) cultivars collected from six different regions have been compaired by exposing them to drought stress^9^. To evaluate the drought response, commonly used parameters for assessing drought tolerance such as growth reduction, photosynthesis reduction, water content reduction, and electrolyte leakage increase were measured. Leaf wilting and grass height were visually assessed and measured with tools^9^. Chlorophyll fluorescence was measured using a fluorometer to evaluate photosynthetic efficiency. To measure leaf water content and electrolyte leakage, a series of manual processes such as weighing after removing moisture or using a pressure chamber were performed. As a result, a new approach based on image analysis has been developed as an alternative to the existing methods and has begun to attract attention among researchers^10^.

The concept of imaging plants goes beyond simply taking photographs of plants. It involves quantitatively measuring a plant's phenotype through the interaction between the plant and light, including photon absorption, reflection, and emission^11^. To execute this imaging-based phenotyping technology, not only biology, but also interdisciplinary understanding and integration of sensor technology, computer vision, mathematics, and electronic engineering are essential. This approach allows for the objective acquisition of anatomical and physiological characteristics of plants through non-contact and non-destructive methods, surpassing human limitations in accuracy, sensitivity, and processing capacity^11^ Büchi et al.^12^ conducted experiments to visually compare the canopy cover area of plant cover obtained using two image analysis tools (Assess 2.0 and Canopeo) with that obtained through visual evaluation, finding a positive relationship between the two evaluations. However, they recognized the need to control environmental constraints such as light and shadow to obtain more objective results from the acquired images and emphasized that there should be an additional process to accurately measure canopy area, as there are limitations in distinguishing plant cover and weeds using only digital image analysis tools^12^.

There are several vegetation indices used in remote sensing to monitor plant growth and assess vegetation health. Two widely used indices are the Normalized Difference Vegetation Index (NDVI) and the Soil Adjusted Vegetation Index (SAVI). The NDVI is a commonly used index for monitoring corn growth, managing farmland, and predicting yield. It is sensitive to variations in the growth of various crops. Osco et al.^13^, Santana et al.^14^, and Wan et al.^15^ have utilized NDVI in their studies related to corn growth, farmland management, and yield prediction. On the other hand, the SAVI is another popular vegetation index that provides more reliable results by minimizing the influence of soil effects. It takes into account the soil background reflectance, making it more appropriate than NDVI in certain situations. Braz et al.^16^ highlighted the advantages of SAVI, particularly its ability to mitigate the impact of soil variations. SAVI is often used in comparison with NDVI or as an alternative index for vegetation assessment^8^.

In recent years, there has been significant progress in optimizing ground-based high-throughput approaches for non-destructive estimation of spectral vegetation indices (SVIs). These SVIs, such as the normalized vegetation index (NDVI), simple ratio (SR), and chlorophyll indices (CI), serve as indicators of green biomass, leaf area index (LAI), chlorophyll levels, and photosynthesis rate. They are useful for assessing the senescence rate during crop maturation^17–21^. The advent of cost-effective, high-resolution multispectral sensors has led to the widespread use of unmanned aerial vehicles (UAVs) in crop physiology research. UAVs offer a practical approach for temporally quantifying spatial traits in large and diverse crop populations, thereby maximizing selection accuracy across various environmental factors. Multispectral sensors and RGB cameras mounted on UAVs have been employed to capture spectral imagery in different light reflectance bands (near-infrared, red, red-edge, green, and blue). These images have been used to detect biomass^22^, LAI^23^, plant density^24^, and photosynthetic activity^25^ in crops such as rapeseed, barley, and wheat. UAV-based multispectral imagery has also been utilized to estimate the rate of emergence and spring survival in wheat^26^.

In addition to UAVs, satellite-based vegetation data have been instrumental in crop growth monitoring^11,27,28^. More recently, UAV-based vegetation indices such as the enhanced vegetation index (EVI) and the normalized difference red edge (NDRE) have been employed to assess the physiological status of wheat and sorghum crops during maturation under drought conditions^11^. Since senescence plays a crucial role in the selection of stay-green genotypes, there is a need to develop accurate methods using cost-effective spectral data to predict senescence rate. Under extreme stress conditions, early senescence resulting from rapid breakdown of plant tissues and macromolecules, such as chlorophyll, can lead to significant yield losses. Borrell et al.^10^ demonstrated the negative impact of high senescence rates on grain yield under water-limited and heat conditions. Senescence is closely related to green leaf area, chlorophyll content, and canopy temperature. Selecting wheat genotypes with improved stay-green traits, characterized by delayed senescence, can enhance cultivar performance under stressed conditions^29,30^.

This study aims for estimating turfgrass green cover by utilizing high-resolution RGB and multispectral images obtained through UAV technology. The specific objectives of this research are to compare and introduce various methods for estimating percent green cover during turfgrass establishment using UAV-derived data, to demonstrate the accuracy and effectiveness of UAV-derived percent green cover estimation by comparing it with ground-level percent green cover data using different vegetative indexes and to find out the most tolerant grass to cold climate.

## Materials and methods

### Grass farming

**Figure 1:**
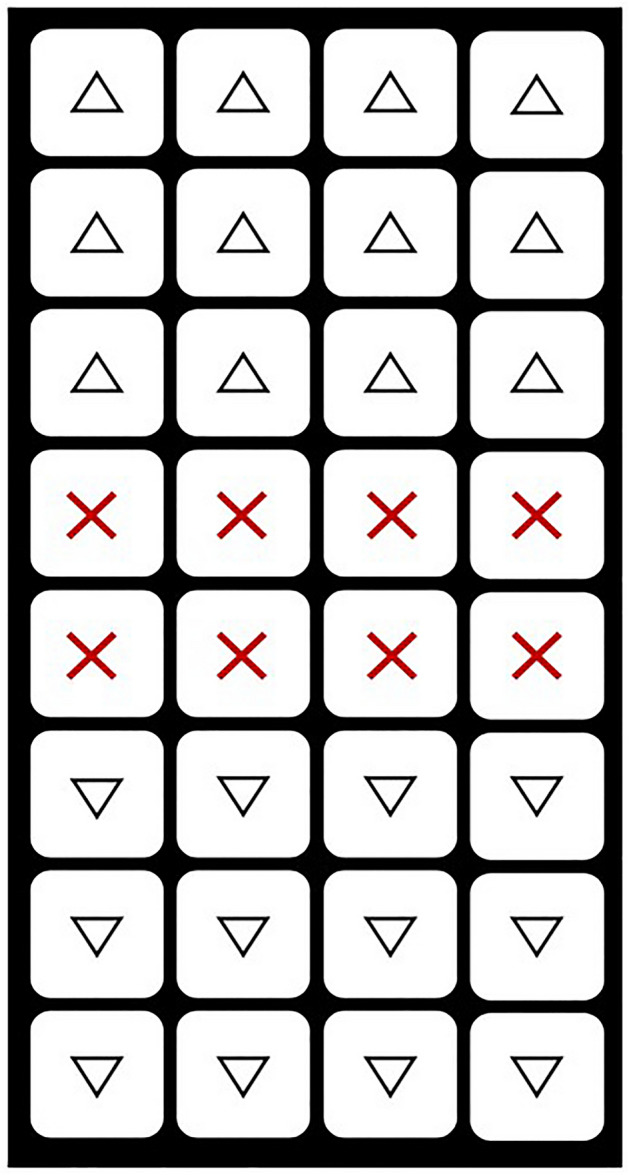
Two germplasm that were different from each other were planted in one pot. △: one germplasm. ▽: the other germplasm that is different △. : no germplasm, it is empty.

**Figure 2:**
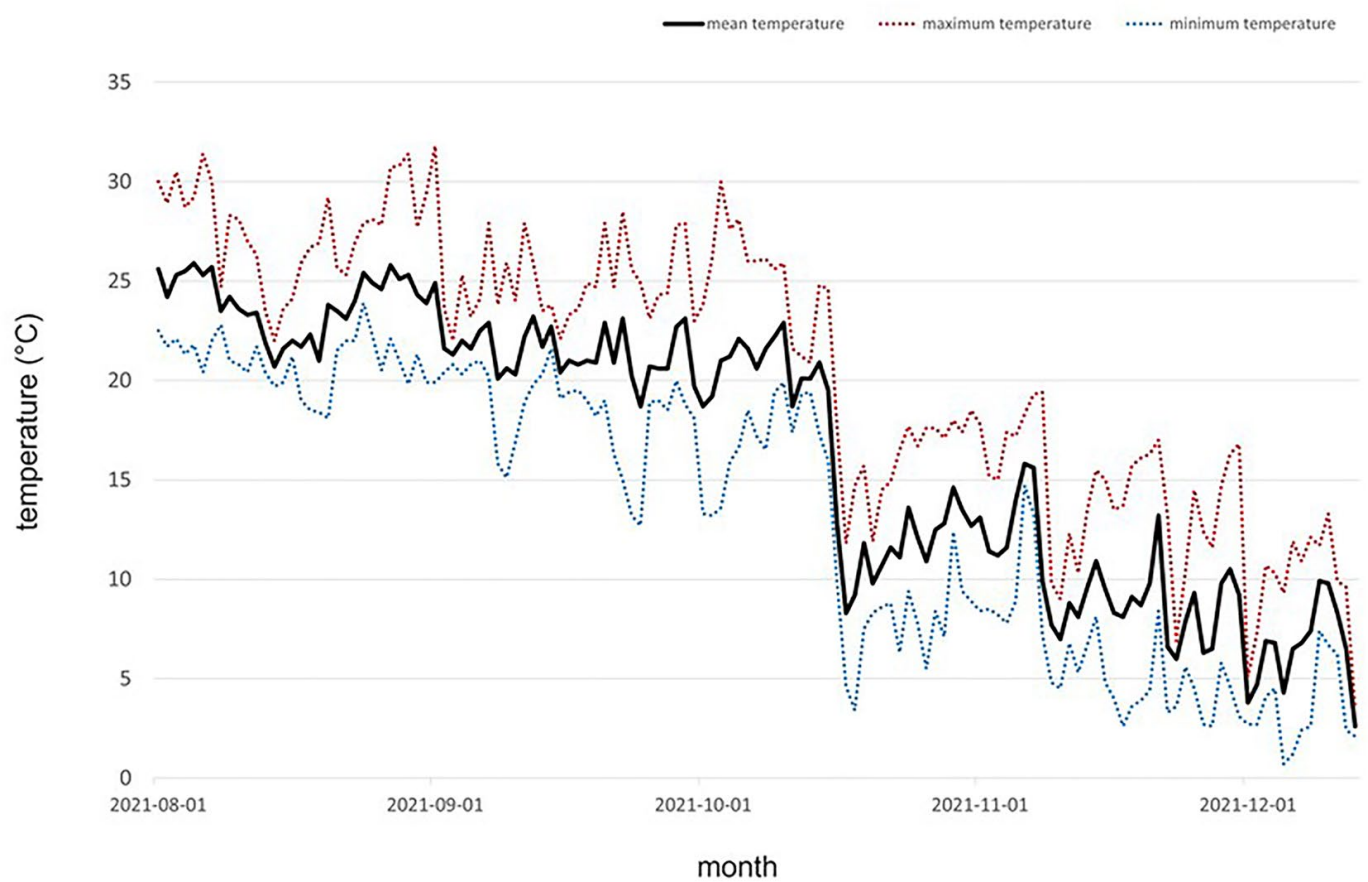
Temperature near the turf greenhouse from where the automatic weather system in Sanchondan is provided.

**Figure 3:**
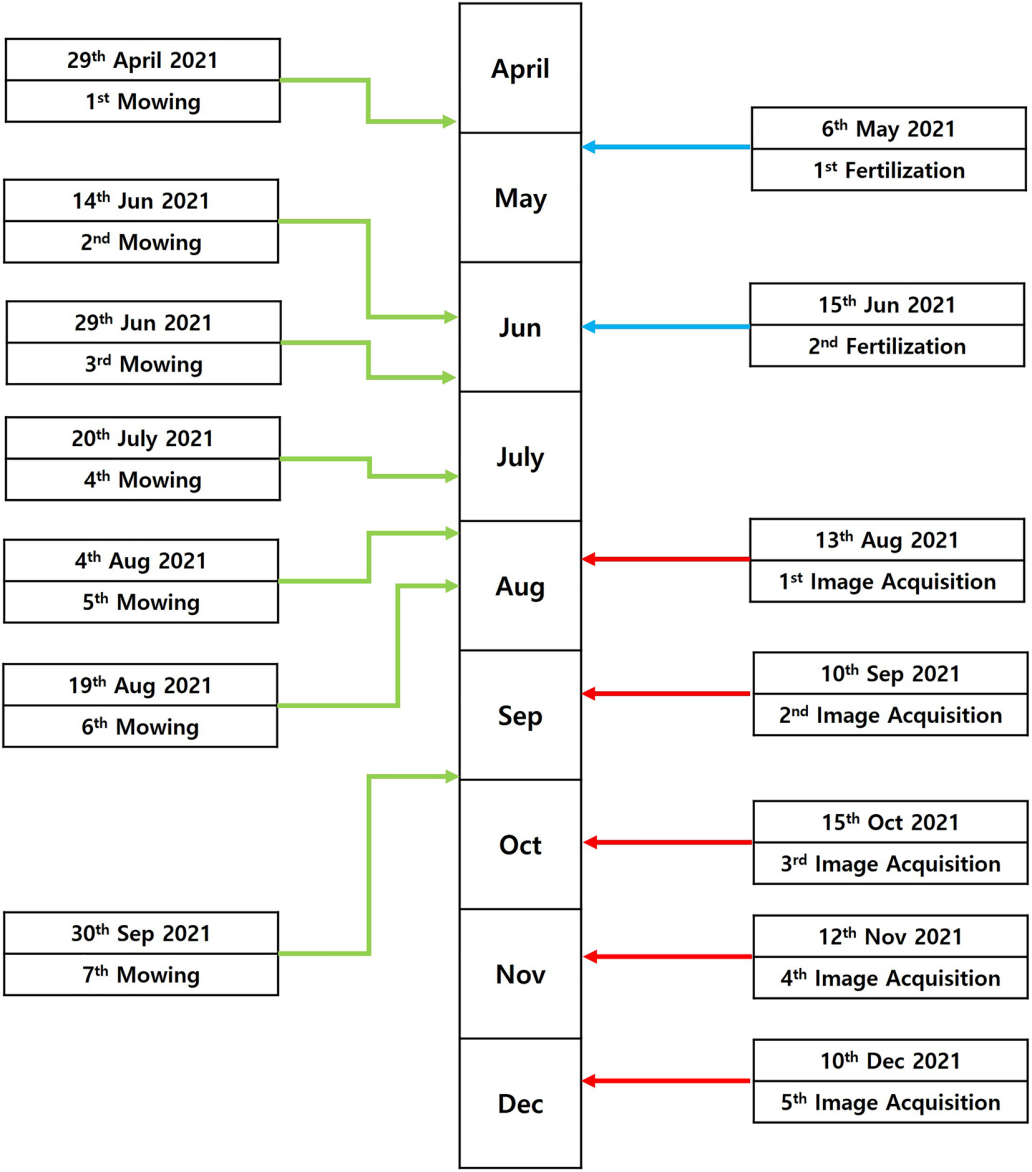
The schedule of mowing and data acquisition in this study.

To conduct this study, a turf cultivation site was created in a facility house located within Jeju National University in Aradong, Jeju City in Jeju Island. The grass used in the experiment was collected from the Forest Life Resources Conservation Field of Jeju National University and transplanted over a period of approximately nine days from July 29th to August 6th, 2020 (Table 1). Thirty-two pot trays (5 cm in height, and 12 cm in length per cell) were used, filled with agricultural biosolid and bedding biosolid. As shown in Fig. 1, one resource was planted in each of the 12 cells on both sides after leaving the middle 8 cells empty. The trays with the planted grass were randomly placed on a bed approximately 70 cm above the ground. Subsequently, watering was carried out using a sprinkler cooler and hose to promote normal grass growth. To maintain the grass, a ventilation fan was installed and the side windows of the facility house were kept open to maintain a temperature similar to the external environment (Fig. 2). In addition, to promote high-density growth of the grass, fertilization was carried out twice in May and June 2021, and seven weeding sessions were performed between April and September 2021 at the appropriate times (Fig. 3). Above all, we managed the grass genetic resources by ensuring that the grass in each designated area did not mix by checking and trimming any overlapping stems every day.

**Table 1:** List of species of Zoysia grass germplasm used in the study.

No	Germplasm	Species	No	Germplasm	Species
1	aewolbudu	–	11	san2daejogu	–
2	san41	*Zoysia tenuifolia* Willd. ex Trin	12	san568	*Zoysia tenuifolia* Willd. ex Trin
3	san303	*Zoysia tenuifolia* Willd. ex Trin	13	san86	*Zoysia japonica* Steud
4	san45	*Zoysia japonica* Steud	14	suncheon	–
5	san351	*Zoysia tenuifolia* Willd. ex Trin	15	san9dangugdae	–
6	san208	*Zoysia tenuifolia* Willd. ex Trin	16	san184	*Zoysia japonica* Steud
7	san177	*Zoysia tenuifolia* Willd. ex Trin	17	san180	*Zoysia japonica* Steud
8	san254	*Zoysia tenuifolia* Willd. ex Trin	18	san135	*Zoysia japonica* Steud
9	san128	*Zoysia japonica* Steud	19	san218	*Zoysia japonica* Steud
10	san398	*Zoysia tenuifolia* Willd. ex Trin	20	san187	*Zoysia japonica* Steud

### Image acquisition

**Figure 4:**
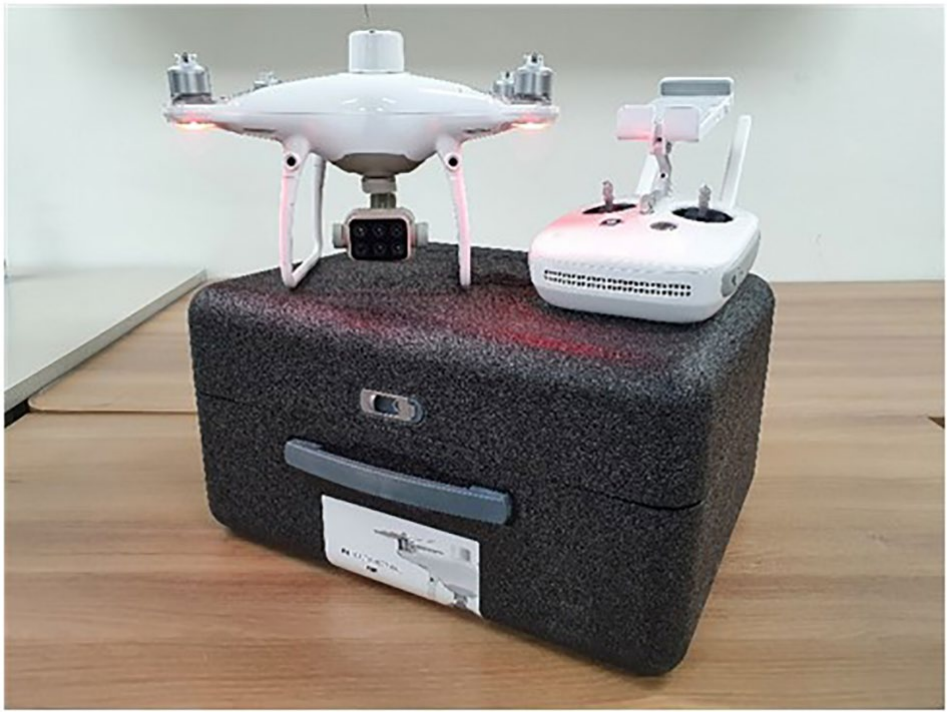
RGB camera sensor and multispectral sensor mounted Phantom 4 Multispectral.

**Figure 5:**
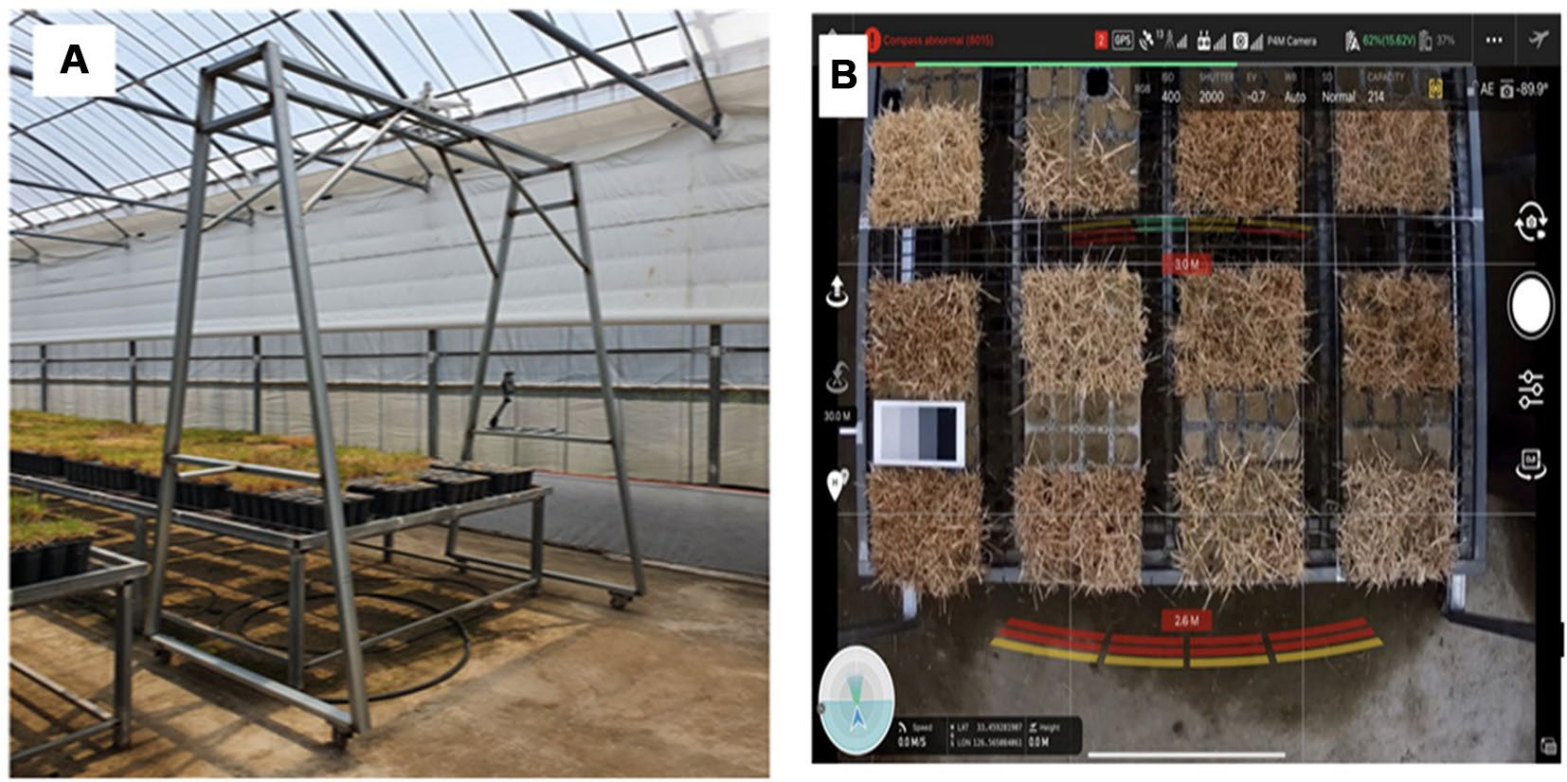
Customized vehicle for turf image acquisition (**A**) and captured DJI GS pro application image (**B**) where radiometric calibration target was located in.

For the one-year-old grass, monthly mid-month (August 13th, September 10th, October 15th, November 12th, and December 10th ) images were taken for five months from August to December 2021. A Phantom 4 Multispectral (WM336, DJI) unmanned aerial vehicle equipped with six 1–2.9″ CMOS sensors (Fig. 4) was used for the experiment, and the wavelength range information for each sensor is shown in Table 2. Prior to the shooting, weed removal and moss covering with sand were performed to prevent other plants from appearing in the images. The Shooting was performed using the DJI GO pro app, the ISO value was set to 400, and the white balance was automatically adjusted. The shooting started at 1 p.m. and lasted for about 2 h. To shoot the grass, a drone with a sensor attached was placed on a custom-made device and kept at a fixed distance vertically 2 m above the grass to ensure that data was acquired with a fixed interval (Fig. 5A). Additionally when moving the device to shoot the grass, a radiation correction target plate (Type 882 Woven polyester fabric, Group 8 Technology, United States) was also moved so that the target plate was visible in all images (Fig. 5B). Images were stored in different file formats depending on the sensor used, with RGB images stored in JPEG format and multi-spectral images stored in TIFF format. The size of all images was 1600 × 1300 pixels, which was the same for all sensors. A total of 285 temporal data points (57 per session) were obtained over five sessions.

**Table 2:** Sensor information of Phantom 4 multispectral.

Band	Central wavelength (nm)	Wavelength width (nm)
Blue	450	32
Green	560	32
Red	650	32
Red edge	730	32
Near-infrared	840	32

### Image preprocessing

There are some preprocessing steps performed for image analysis. First, distortion correction was performed to flatten out the radial distortion caused by camera lens characteristics. Second, the radiometric correlation was necessary to equalize the light conditions for all acquired images, as uneven lighting conditions can affect the accurate characterization of plant features^31,32^. Third, histogram equalization was executed to improve the contrast of the RGB image, which had become dark after radiometric correction, making it difficult to discern the color of the grass^33^. Fourth, image alignment was performed on the NIR and Red images used to derive NDVI. This was necessary because of the slight physical differences in the camera positions used in this study, which caused the two images to be captured with slightly different positions. Therefore, the Red image had to be shifted by the number of pixels that differed from the NIR image to align the two images.

#### Distortion correction

**Figure 6:**
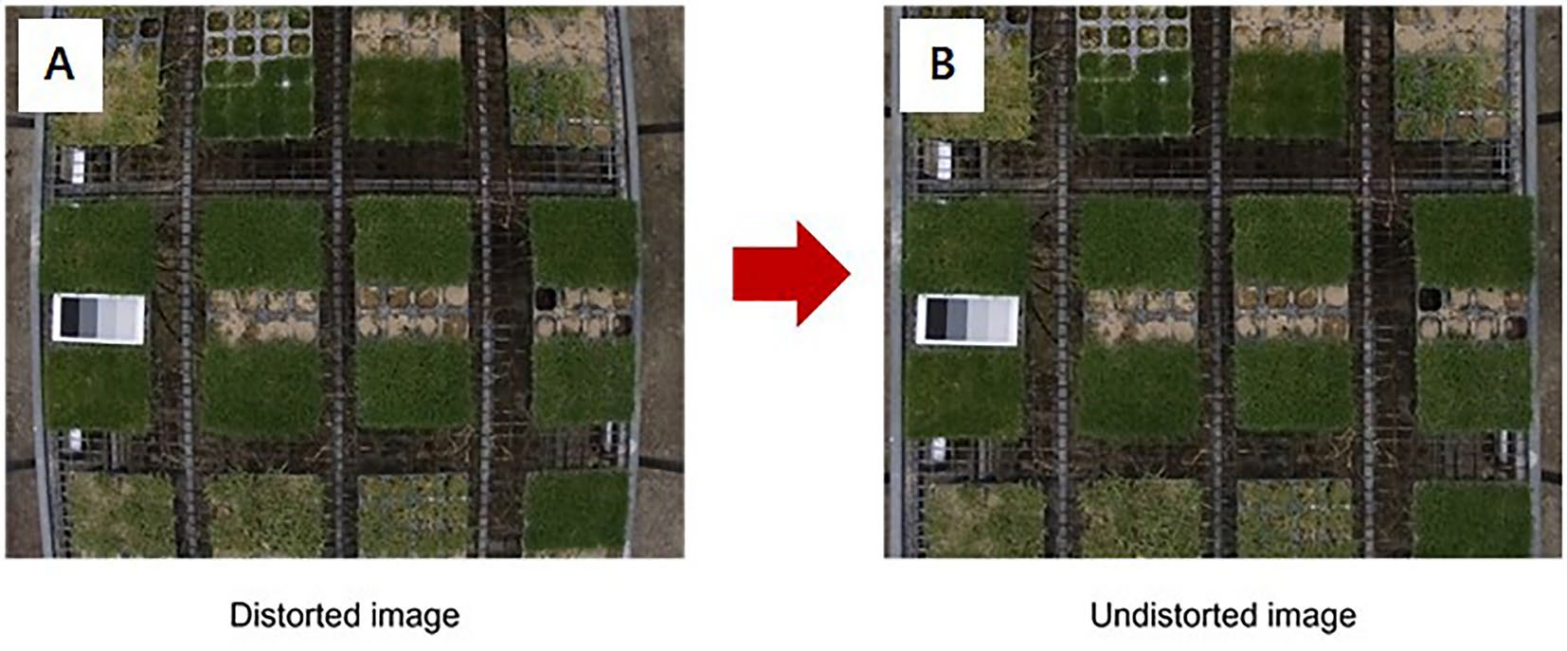
Distortion correction (**A**) distorted image (**B**) undistorted image.

The first is distortion correction, where the acquired image shows a radial distortion in the form of a convex bulge in the center (Fig. 6A). To solve this problem, initial processing was performed using the software Pix4D Mapper (Pix4D SA, Switzerland) to correct the camera lens distortion (Fig. 6B).

#### Radiometric calibration and histogram equalization

**Figure 7:**
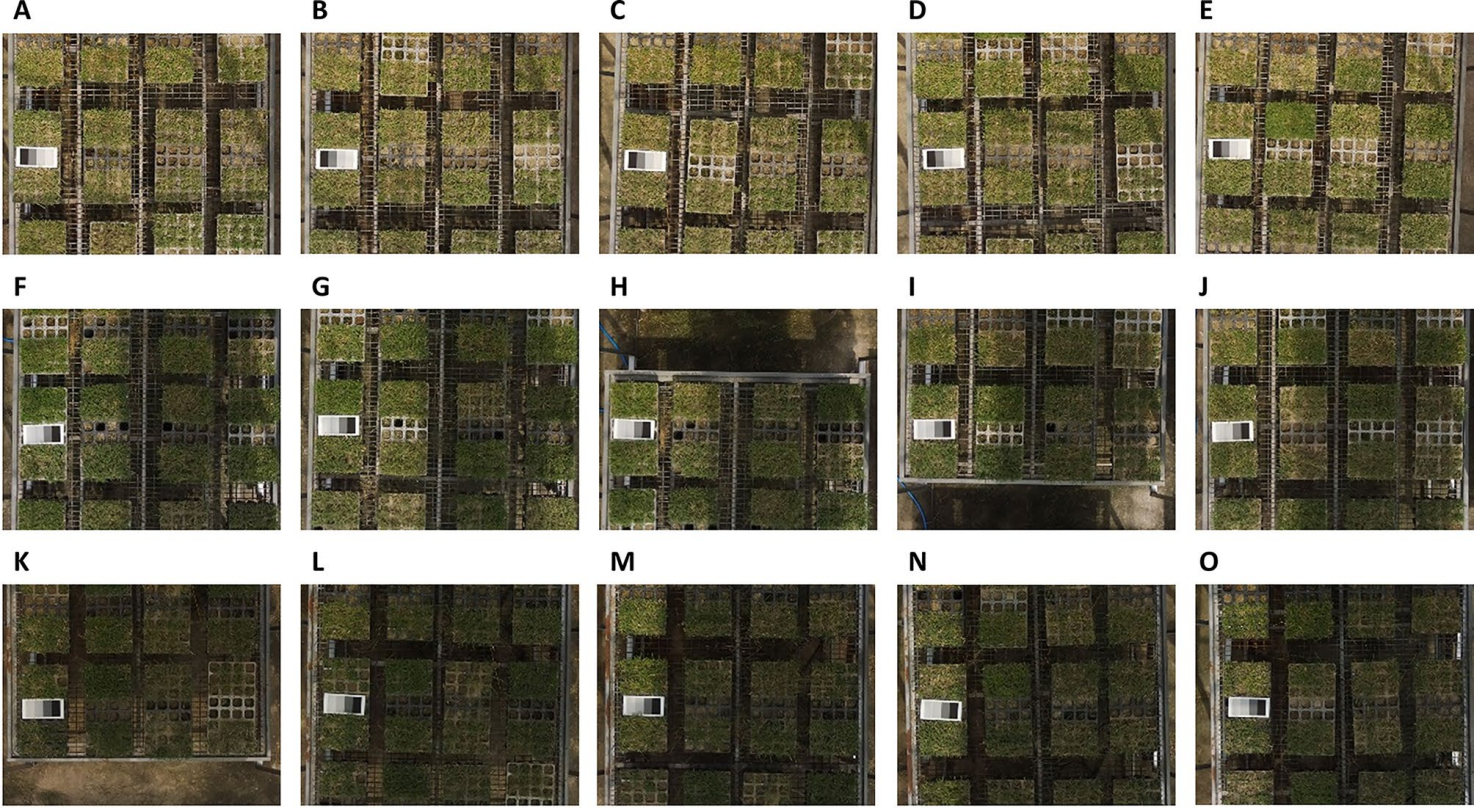
The affect of light conditions image data in the same day. (**A**–**O**) are the image data from the same day.

The light conditions vary from day to day, even for data acquired on the same day, depending on the weather (Fig. 7). The process required to equalize the light conditions is radiometric calibration. It uses a digital numerical value for the image and a fixed reflectance value. It converts every digital number in the image to the reflectance of an object’s surface. This is calculated using the best-fit equation for each band of a homogeneous object. In this study, a professional radiometric calibration reference tarp with uniform reflectance was used, and the surface reflectance value of the radiometric calibration tarp was provided by Group 8 Technology manufacturing company. The camera responses of the RGB and multispectral bands, which are necessary information for radiometric calibration, were obtained from the work of Burggraaff et al.^31^ and Lu et al.^34^, respectively. Finally, the exponential model for radiometric calibration of RGB and the linear model for that of multispectral images were developed by using the empirical line calibration equation^35^. With this information, we obtained digital numerical values for each image and proceeded with the radiometric correction.

**Figure 8:**
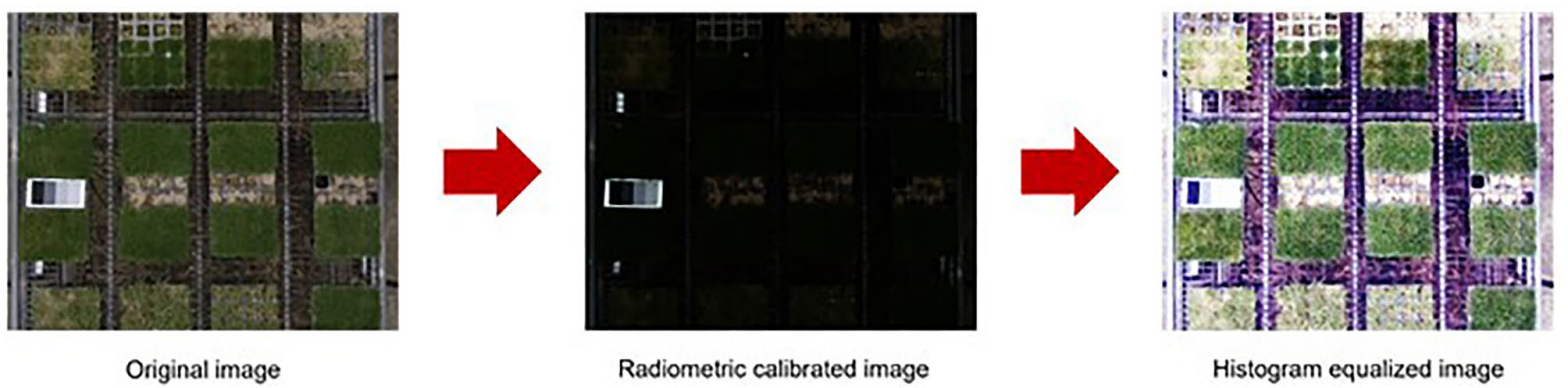
Radiometric calibration of RGB image and hisgogram equalization of it in order.

In addition, it was applied for RGB images with radiometric calibration to perform histogram equalization which straightens the histogram of pixels whose brightness values are clustered towards the dark side of the image (Fig. 8) using MATLAB (R2015b, MathWorks).

#### Image alignment

**Figure 9:**
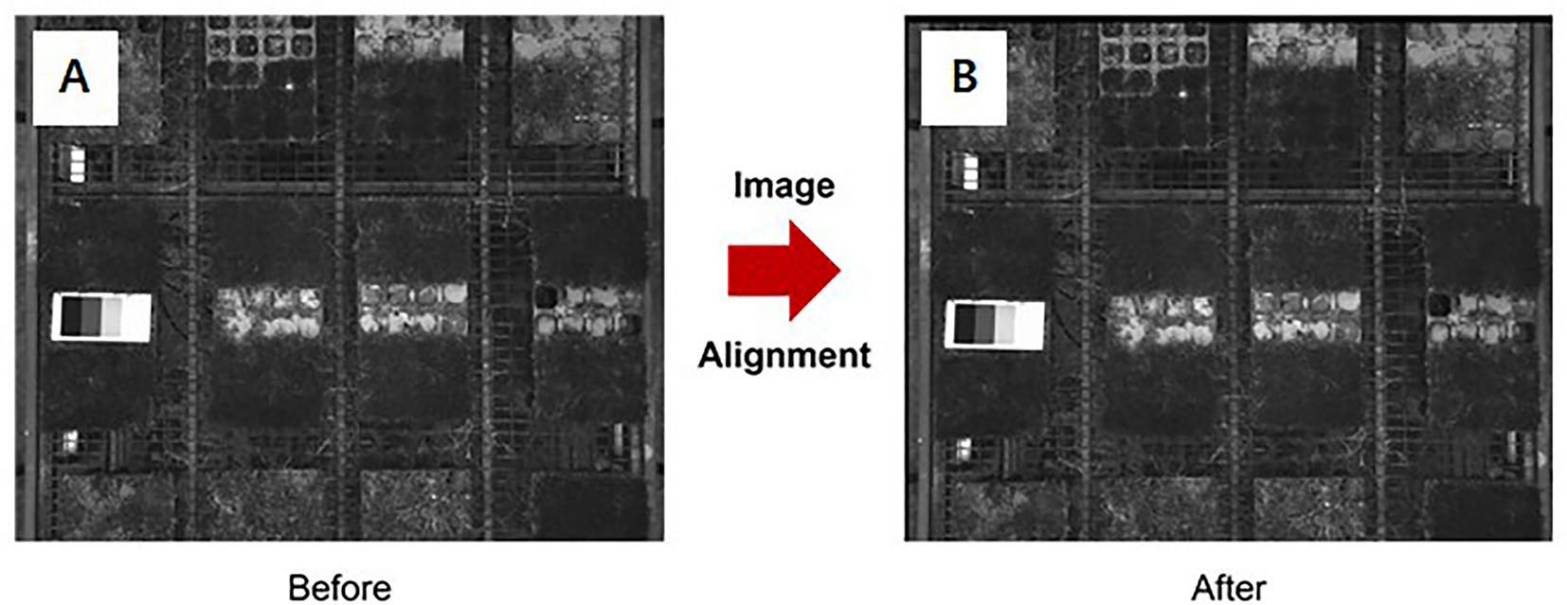
Image alignment of RED band image into NIR band image (**A**) before, (**B**) after.

For the multispectral image calibrated, image alignment was performed to match the NIR band image by shifting the RED band image based on the NIR (Near Infrared) band image (Fig. 9).

Their metadata was used to determine the difference in pixels between the RED and NIR band images in rows and columns. The metadata includes information on the Relative Optical Center X and Y pixel positions, which indicate the physical distance from the NIR lens. In this case, the values for both are zero. It was exported from the raw data and used ‘pyexiv2’ library to convert a TIFF file to an XMP file in Python. The ‘pyexiv2’ library is a Python binding for the Exiv2 library, which is a C +  + library for manipulating image metadata.

The image was processed using in-house software written in Python 3.7.10. The software was used to extract the digital numerical values of the images, apply the radiometric calibration equation, and align the positions of the multispectral images.

### Image analysis

#### GCP (green cover percentage) computation using RGB images

To calculate the greenness rate of the grass for each round, it is necessary to know the total canopy area and green cover area of each grass and calculate it using the formula below (Eq. 1).

1 $${\text{GCP}}\;\left( \% \right) = \left( {{\text{Green}}\;{\text{Cover}}\;{\text{Area}}\;{\text{of}}\;{\text{each}}\;{\text{grass}}} \right){/}\left( {{\text{Total}}\;{\text{Canopy}}\;{\text{Area}}} \right) \times {1}00$$

**Figure 10:**
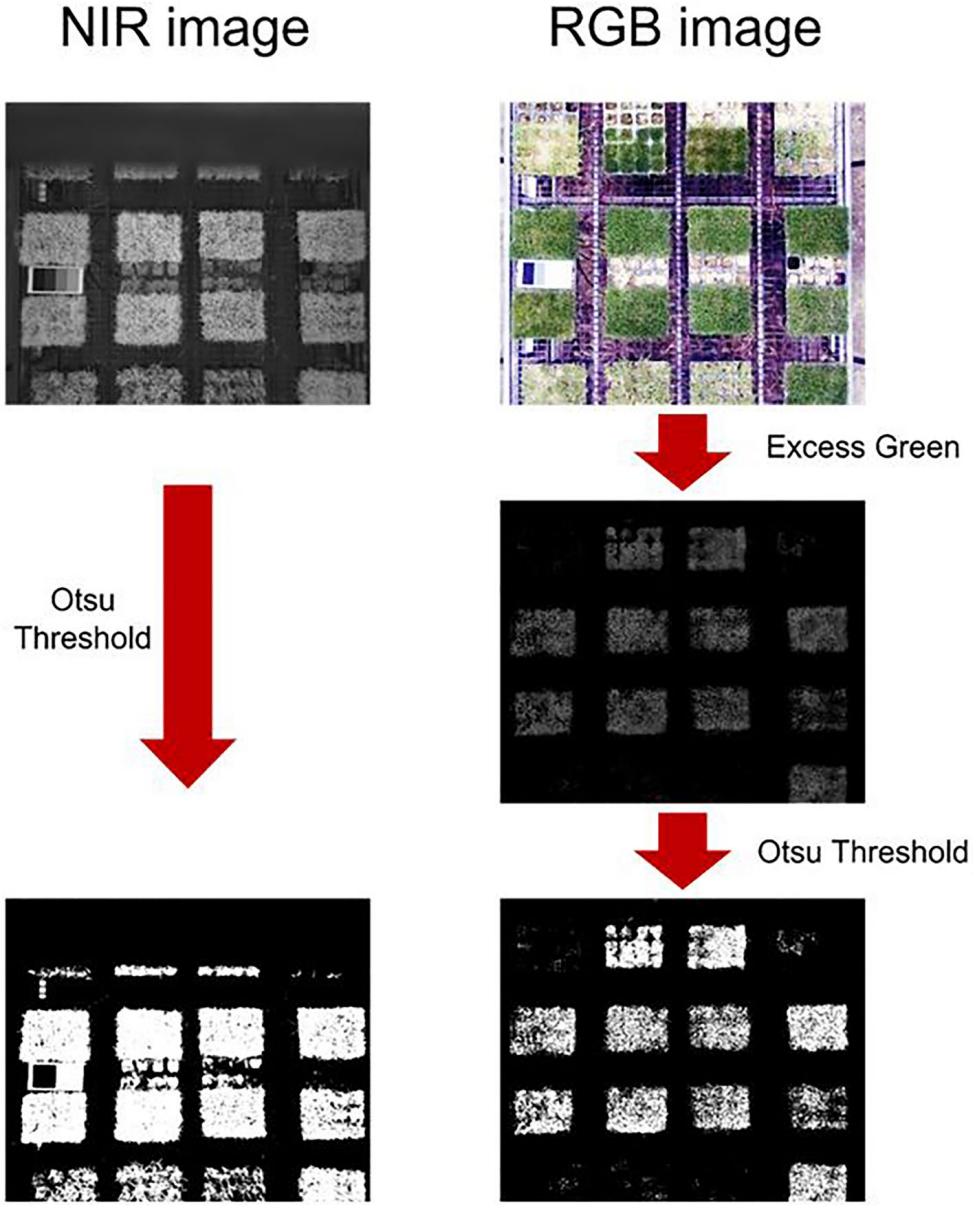
Applying Otsu threshold both NIR images and RGB images processed with Excess Green method.

**Figure 11:**
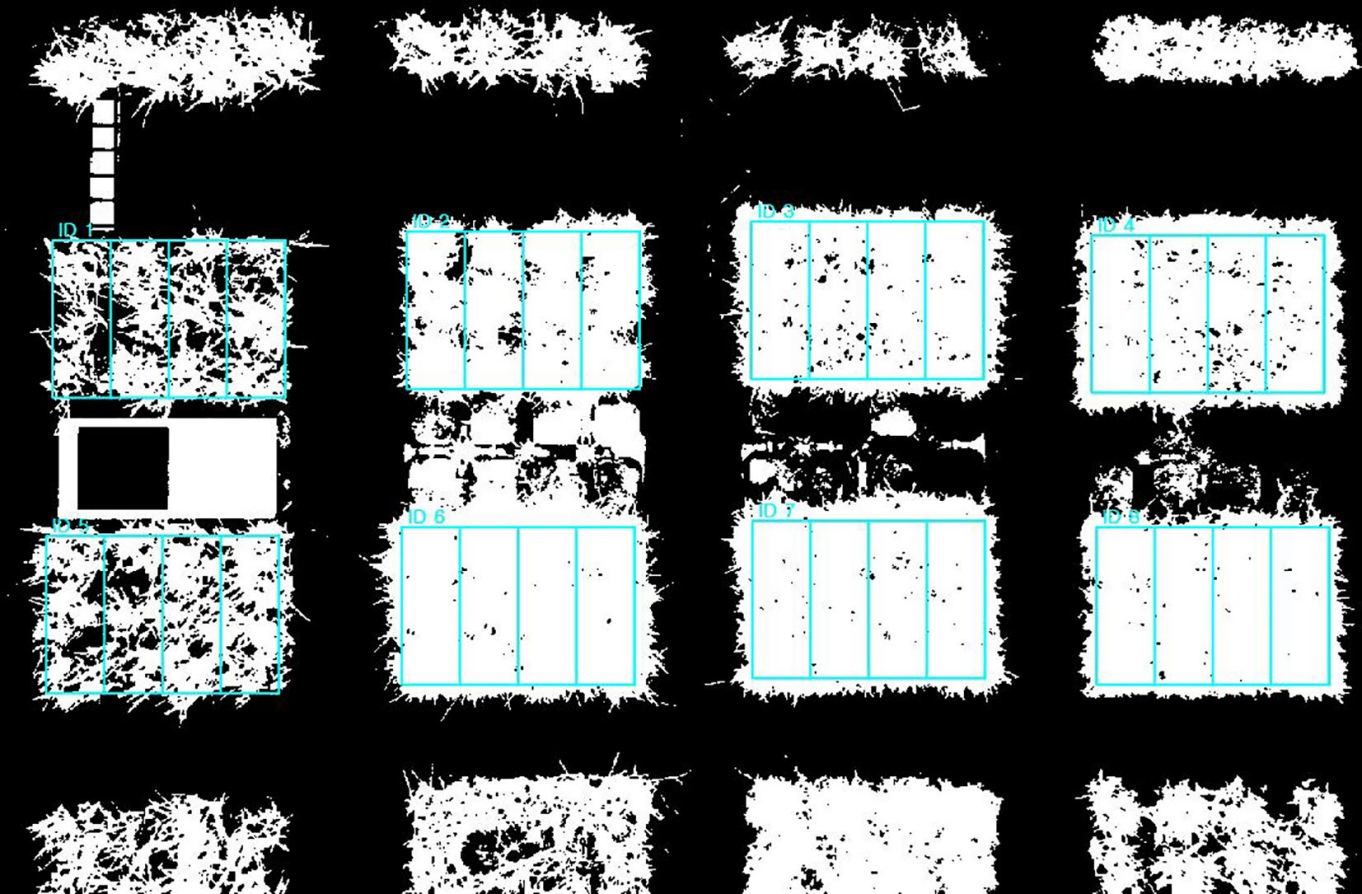
Region of interest (ROI) in NIR images processed Otsu method for extracting grass canopy.

First, the total canopy area of the grass was obtained using NIR images. NIR images show a better contrast between plants and soil than other images, making it easier to identify the boundaries of plant leaves^36^. The green area of the plant was extracted using a color-based index, ExG (Excess Green), on a RGB images that had undergone histogram equalization, in MATLAB. Only the green areas were extracted. Finally, to separate the grass and soil, Otus threshold method was applied to the NIR images and the RGB images with ExG applied, in MATLAB. The method divided the pixels in the images into two categories, black and white, and in this experiment, the black pixels represented the background and the white pixels represented the grass or the green area of the grass (Fig. 10). Subsequently, four rectangular regions of interest (ROI) with a size of 48.5 × 132 pixels were repeatedly specified in the grass area of the image, and the number of white pixels inside each ROI was counted to determine the total area and green area of the grass as shown in Fig. 11. This process was performed using self-developed software based on Python for this experiment^51^.

#### Various vegetation indexes computation using multispectral images

In this study, to monitor the degree of stress in the grass over time, we selected the NDVI (Normalized Difference Vegetation Index), which is the most widely used vegetation index. This index is based on the basic principle that healthy vegetation absorbs a lot of light in the visible red region and reflects a lot of light in the near-infrared region, which can be used to determine leaf area index, chlorophyll content, photosynthetic absorbed radiation, growth status, and vitality of vegetation.To calculate the NDVI of the grass, the radiometrically corrected RED and NIR images were used in the equation below (Eq. 2).

2 $${\text{NDVI}} = \left( {{\text{NIR}} - {\text{RED}}} \right)/\left( {{\text{NIR}} + {\text{RED}}} \right)$$

Also, we use the Normalized Difference Red Edge Index (NDRE), Soil-adjusted Vegetation Index (SAVI), and Enhanced Vegetation Index (EVI) to monitor the grass over time. The equations below were used to calculate each VIs (Vegetation Indexes).

3 $${\text{NDRE}} = \left( {{\text{NIR}}{-}{\text{Red}}\;{\text{Edge}}} \right)/\left( {{\text{NIR}} + {\text{Red}}\;{\text{Edge}}} \right)$$

4 $${\text{SAVI}} = \left( {\left( {{\text{NIR }}{-}{\text{ R}}} \right){/}\left( {{\text{NIR}} + {\text{R}} + {\text{L}}} \right)} \right)*\left( {{1} + {\text{L}}} \right)$$

[L is the soil brightness correction factor defined as 0.5 to accommodate most land cover types.]

5 $${\text{EVI}} = {\text{G}}*\left( {\left( {{\text{NIR }}{-}{\text{ R}}} \right)/\left( {{\text{NIR}} + {\text{C1}}*{\text{R}}{-}{\text{C2}}*{\text{B}} + {\text{L}}} \right)} \right)$$

[G is the gain factor, which is typically set to 2.5; C1 and C2 are coefficients used to reduce atmospheric influences, which commonly used values are C1 = 6 and C2 = 7.5; L is the canopy background adjustment factor, which is commonly used value is L = 1.]

**Figure 12:**
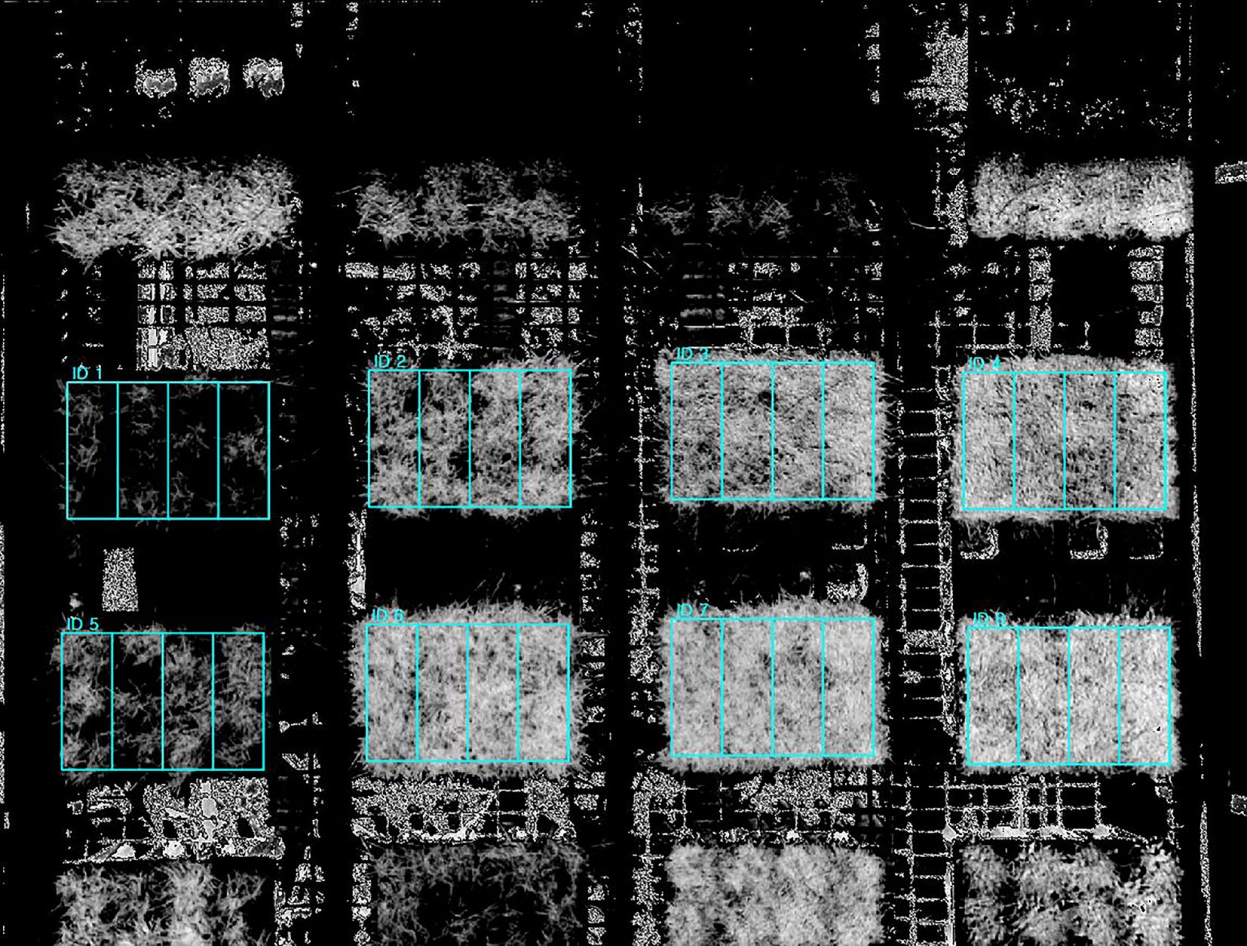
Region of interest (ROI) in NDVI images to divide each box (germplasm) into four replications and computate the average NDVI value for each of them.

After that, as in the case of selecting the ROI for the greening rate in the grass area in the VI images, we selected four ROIs of the same size as before and excluded the background to extract the VIs values of the pixels corresponding to the grass (Fig. 12). The VIs for each grass was then represented by the average value of the extracted VIs values This process was also conducted using software based on Python, which was independently developed for this experiment.

### Statistics analysis

We used Excel (version 2205, Microsoft) to uniformly process values greater than 100% in the greening rate to 100% prior to statistical analysis. This was done to address an issue where during the process of removing moss, some areas were not fully removed, leading to a slight difference in the location of the green area within the grass, resulting in experimental errors in the calculation of the greening rate.

We performed statistical analysis on the temporal data of the greening rate, NDVI, NDRE, SAVI, and EVI values using the R programming language. Since the data did not meet the assumption of normality, nonparametric Kruskal–Wallis tests were used to determine statistical significance. Subsequently, post hoc analysis was conducted using the rank-based Dunn test, and *p*-values were adjusted using the Benjamini-Hochberg (BH) method. The correlation between the greening rate and NDVI, NDRE, SAVI, and EVI for each session was analyzed using the Pearson correlation coefficient.

## Results

**Figure 13:**
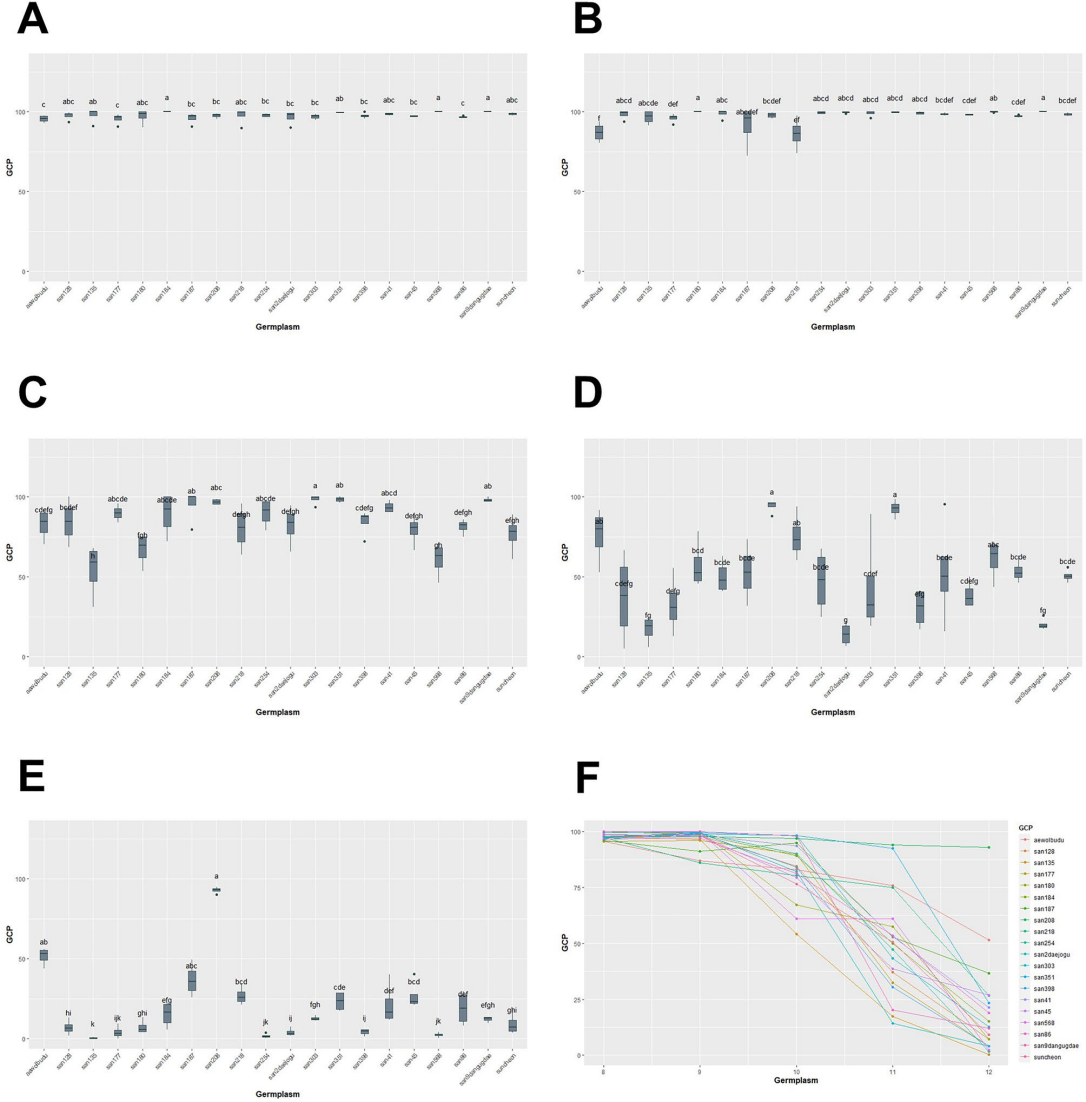
GCP value of turf germplasms from August to December and Transition of GCP values by the months. (**A**) August, (**B**) September, (**C**) October, (**D**) November, (**E**) December, (**F**) Transiton of GCP value during August to December.

**Figure 14:**
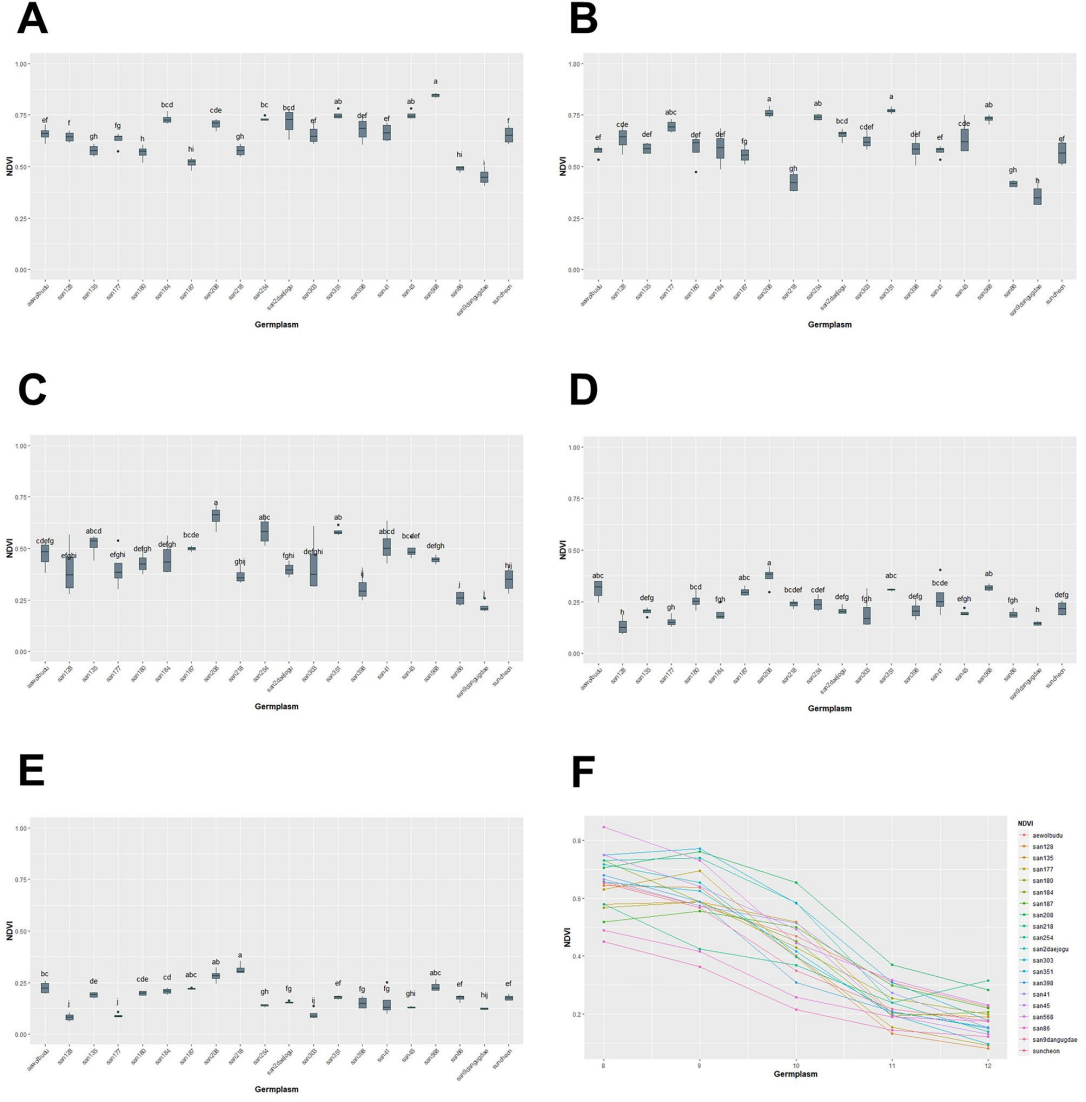
NDVI value of turf germplasms from August to December and Transition of NDVI values by the months. (**A**) August, (**B**) September, (**C**) October, (**D**) November, (**E**) December, (**F**) Transiton of NDVI value during August to December.

**Figure 15:**
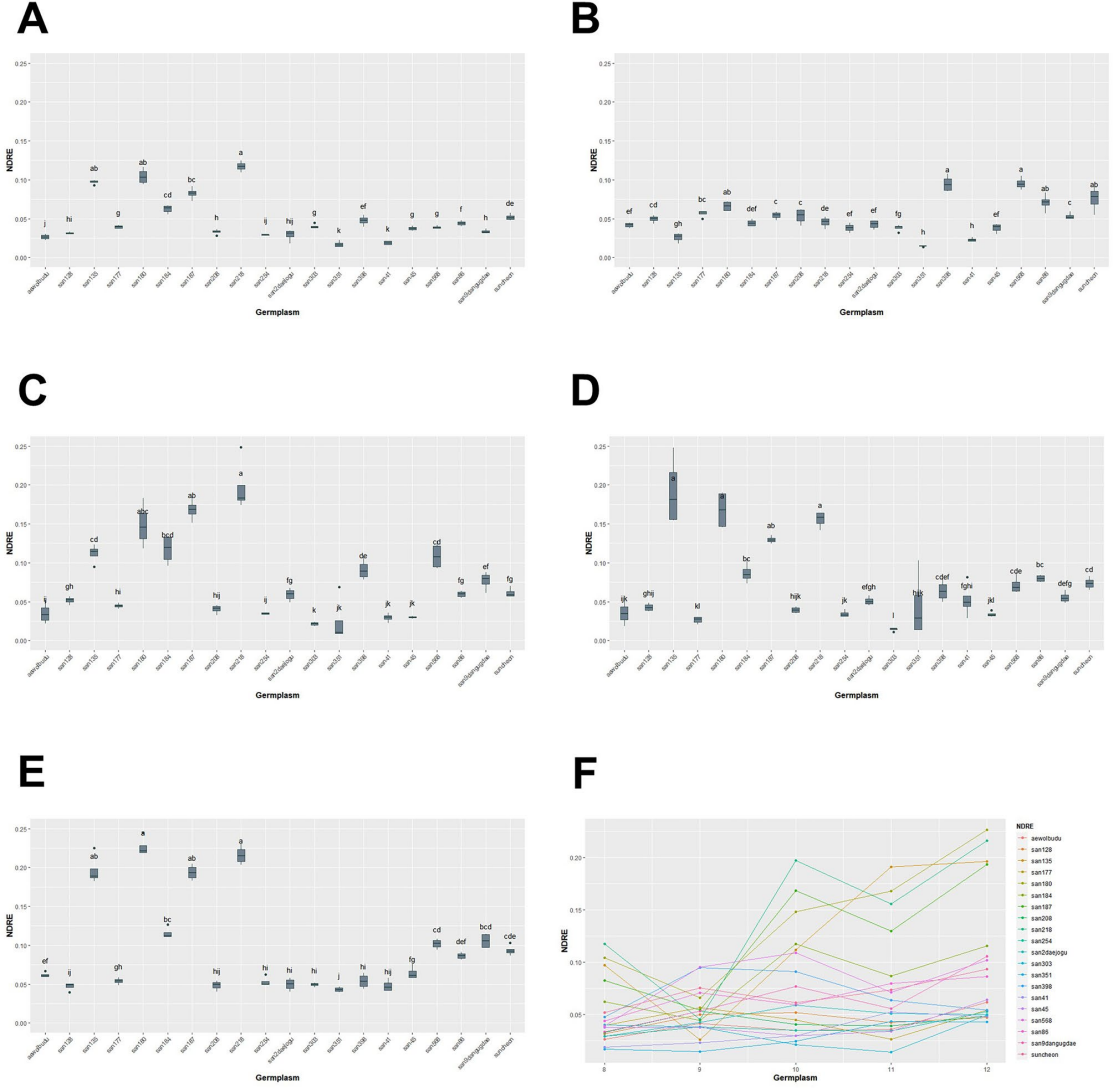
NDRE value of turf germplasms from August to December and Transition of NDRE values by the months. (**A**) August, (**B**) September, (**C**) October, (**D**) November, (**E**) December, (**F**) Transiton of NDRE value during August to December.

**Figure 16:**
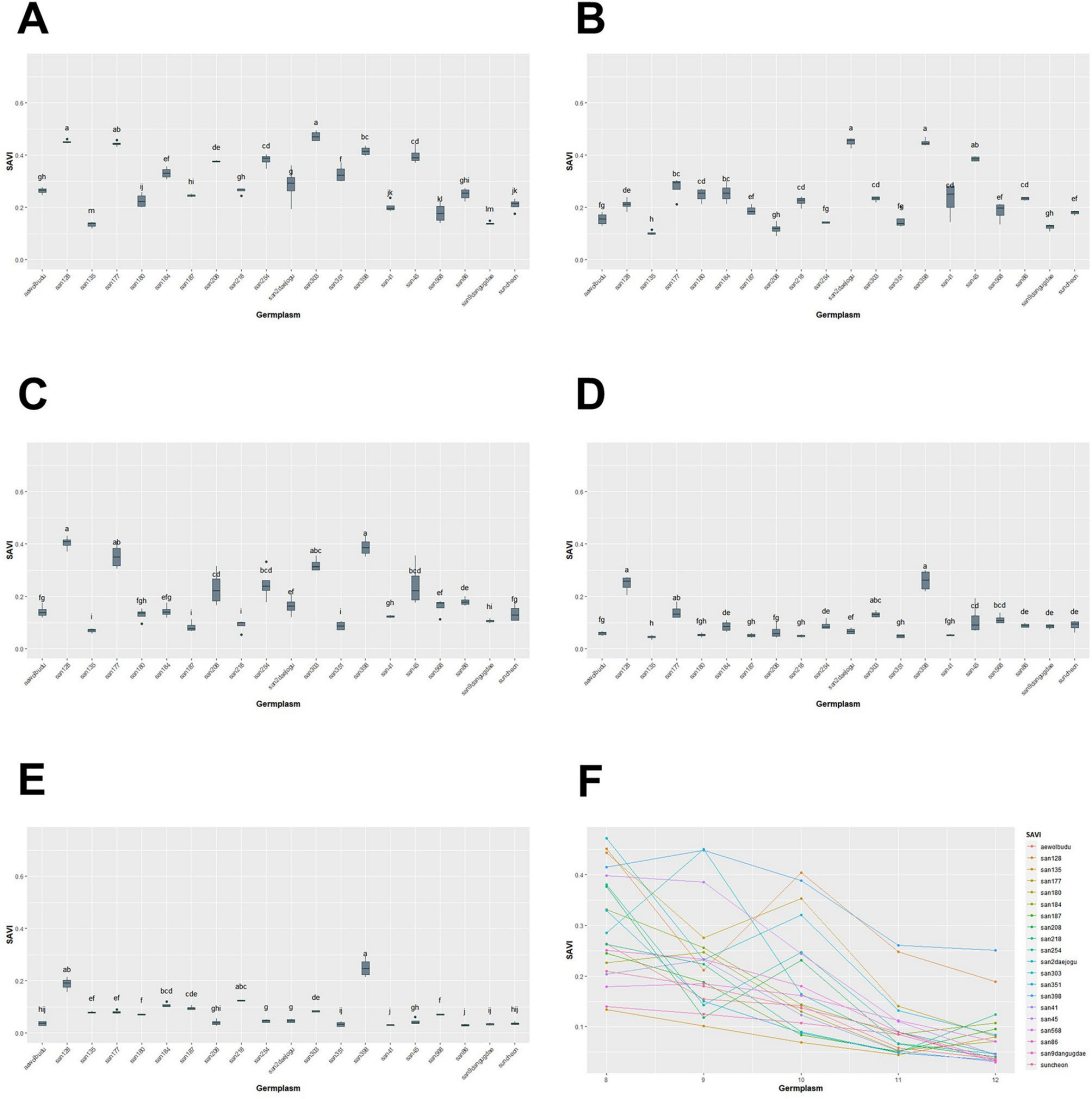
SAVI value of turf germplasms from August to December and Transition of SAVI values by the months. (**A**) August, (**B**) September, (**C**) October, (**D**) November, (**E**) December, (**F**) Transiton of SAVI value during August to December.

**Figure 17:**
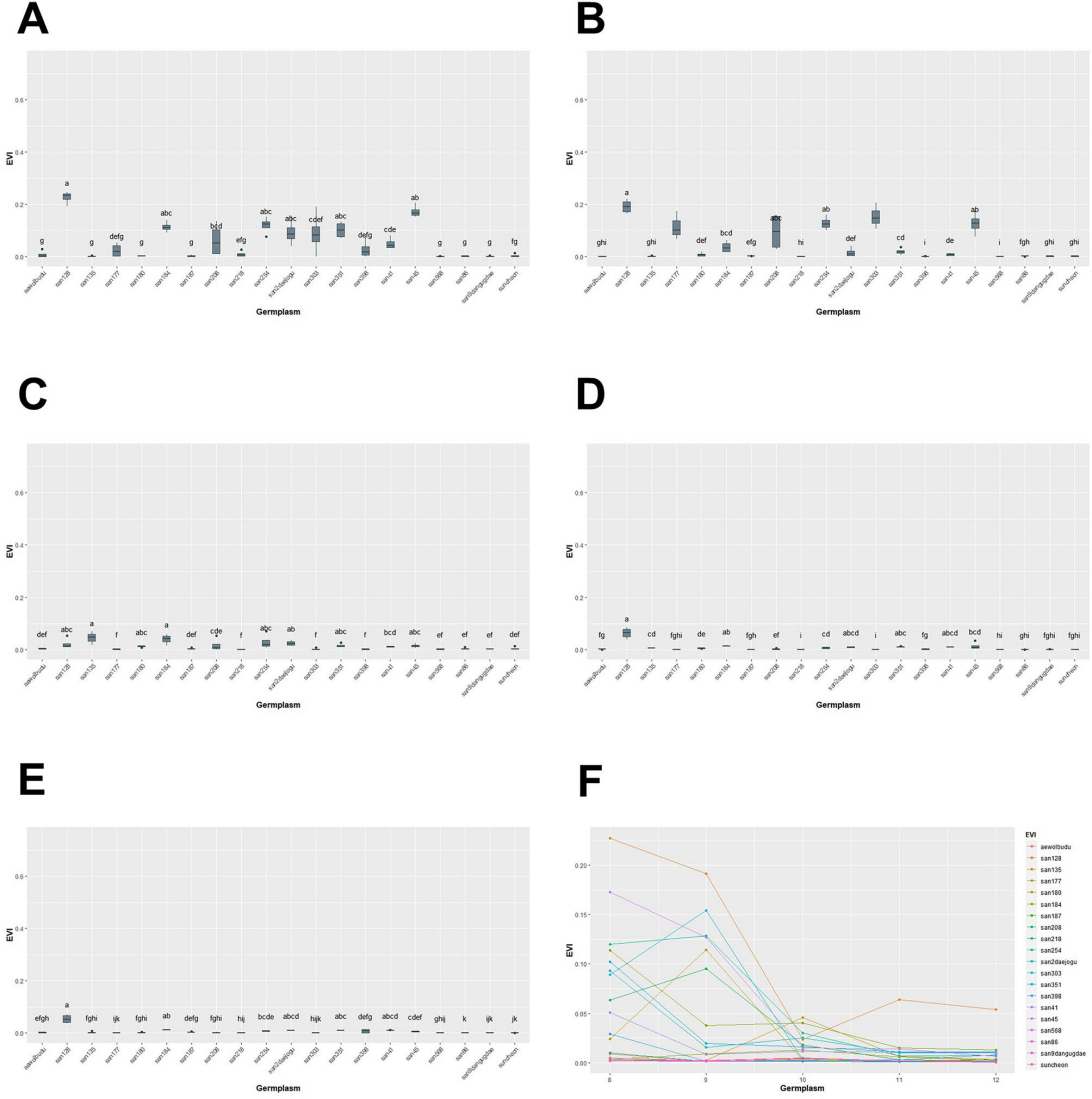
EVI value of turf germplasms from August to December and Transition of EVI values by the months. (**A**) August, (**B**) September, (**C**) October, (**D**) November, (**E**) December, (**F**) Transiton of EVI value during August to December.

We conducted a study between August and December, where we measured GCP, NDRE, NDVI, SAVI, and EVIfor five months. Our analysis using the Kruskal–Wallis test showed that there were statistically significant differences in GCP and VIs data for each genetic resource at every measurement point, with a *p*-value of less than 0.05 (Table 3, Figs. 13, 14, 15, 16, 17). We presented the changes in GCP and NDVI for each genetic resource in Tables 4 and 5, respectively. These tables illustrate how the rankings of genetic resources differed and how browning rates varied among resources for each measurement period. We also observed differences in the size of the variance in GCP and NDVI between repetitions for each genetic resource by month. Until October, the GCP was relatively consistent across all genetic resources. However, starting in October, we began to see differences among genetic resources, with the differences becoming most pronounced in November. In contrast, the NDVI showed a certain degree of consistency among genetic resources, as shown in Fig. 14.

**Table 3:** Kruskal–Waliis test results for GCP and NDVI, NDRE, SAVI and EVI data.

Month		GCP		NDVI		NDRE		SAVI		EVI	
	df	χ2	*p* value	χ2	*p* value	χ2	*p* value	χ2	*p* value	χ2	*p* value
Agu	19	42.24	< 0.01	69.05	< 0.001	76.11	< 0.001	75.04	< 0.001	57.87	< 0.001
Sep	19	44.77	< 0.001	63.96	< 0.001	71.31	< 0.001	70.50	< 0.001	67.23	< 0.001
Oct	19	51.79	< 0.001	58.24	< 0.001	73.24	< 0.001	71.03	< 0.001	59.44	< 0.001
Nov	19	54.29	< 0.001	58.21	< 0.001	68.98	< 0.001	67.57	< 0.001	67.64	< 0.001
Dec	19	68.69	< 0.001	70.28	< 0.001	72.89	< 0.001	72.84	< 0.001	63.39	< 0.001

**Table 4:** Average difference of GCP among germplasm for August to December.

Aug		Sep		Oct		Nov		Dec	
Germplasm	Measured value	Germplasm	Measured value	Germplasm	Measured value	Germplasm	Measured value	Germplasm	Measured value
san9dangugdae	100 ± 0.00 *a	san9dangugdae	100 ± 0.00 a	san303	98 ± 3.11 a	san208	94 ± 4.16 a	san208	92 ± 2.05 a
san568	100 ± 0.00 a	san180	100 ± 0.00 a	san351	98 ± 1.66 ab	san351	93 ± 5.34 a	aewolbudu	51 ± 5.52 ab
san184	100 ± 0.00 a	san568	99 ± 0.21 ab	san9dangugdae	98 ± 1.37 ab	aewolbudu	76 ± 17.12 ab	san187	36 ± 10.28 abc
san351	99 ± 0.27 ab	san184	98 ± 2.75 abc	san187	94 ± 10.26 ab	san218	75 ± 14.27 ab	san218	26 ± 5.77 bcd
san135	97 ± 4.49 ab	san351	99 ± 0.37 abcd	san208	96 ± 1.78 abc	san568	61 ± 12.82 abc	san45	26 ± 9.08 bcd
suncheon	98 ± 0.72 abc	san2daejogu	99 ± 0.50 abcd	san41	93 ± 3.58 abcd	san180	57 ± 14.81 bcd	san351	23 ± 6.13 cde
san41	98 ± 0.69 abc	san254	99 ± 0.73 abcd	san254	90 ± 9.07 abcde	san41	53 ± 32.54 bcde	san41	21 ± 13.01 def
san128	97 ± 2.72 abc	san398	99 ± 0.87 abcd	san177	89 ± 5.01 abcde	san254	47 ± 20.11 bcde	san86	18 ± 10.57 def
san218	97 ± 5.04 abc	san303	98 ± 1.96 abcd	san184	89 ± 13.46 abcde	san86	54 ± 7.07 bcde	san184	15 ± 8.08 efg
san180	96 ± 4.74 abc	san128	98 ± 2.92 abcd	san128	84 ± 13.80 bcdef	suncheon	51 ± 3.86 bcde	san9dangugdae	12 ± 1.74 efgh
san254	97 ± 0.96 bc	san135	96 ± 4.18 abcde	san398	84 ± 8.06 cdefg	san184	50 ± 10.21 bcde	san303	12 ± 1.47 fgh
san398	97 ± 1.76 bc	san187	91 ± 13.01 abcdef	aewolbudu	82 ± 9.80 cdefg	san187	53 ± 17.65 bcde	suncheon	9 ± 6.74 ghi
san208	97 ± 1.58 bc	san41	98 ± 0.85 bcdef	san45	79 ± 9.38 defgh	san303	43 ± 31.57 cdef	san180	7 ± 4.16 ghi
san45	97 ± 0.50 bc	suncheon	98 ± 0.92 bcdef	san2daejogu	82 ± 12.33 defgh	san45	39 ± 8.41 cdefg	san128	7 ± 4.64 hi
san303	96 ± 1.66 bc	san208	97 ± 1.75 bcdef	san86	81 ± 4.67 defgh	san128	37 ± 27.65 cdefg	san398	4 ± 2.04 ij
san2daejogu	96 ± 4.30 bc	san45	98 ± 0.30 cdef	san218	80 ± 14.10 defgh	san177	32 ± 17.71 defg	san2daejogu	3 ± 2.40 ij
san187	95 ± 3.55 bc	san86	97 ± 0.80 cdef	suncheon	76 ± 11.66 efgh	san398	31 ± 12.28 efg	san177	4 ± 3.99 ijk
san86	96 ± 0.54 c	san177	95 ± 2.69 def	san180	67 ± 10.53 fgh	san9dangugdae	20 ± 3.82 fg	san568	3 ± 1.53 jk
aewolbudu	95 ± 2.14 c	san218	86 ± 9.86 ef	san568	60 ± 10.93 gh	san135	17 ± 8.31 fg	san254	2 ± 1.53 jk
san177	95 ± 3.30 c	aewolbudu	87 ± 5.99 f.	san135	54 ± 16.74 h	san2daejogu	14 ± 7.39 g	san135	1 ± 0.51 k

*Means of ± standard deviation followed by different letters within columns are significantly different by Dunn test with Benjamini–Hochberg (BH) adjustment. Non-parametric rank data were used for statistical analysis; however, untransformed data are presented.

**Table 5:** Average differences of NDVI among germplasm for August to December.

Aug		Sep		Oct		Nov		Dec	
Germplasm	Measured value	Germplasm	Measured value	Germplasm	Measured value	Germplasm	Measured value	Germplasm	Measured value
san568	0.85 ± 0.01 *a	san351	0.76 ± 0.02 a	san208	0.65 ± 0.06 a	san208	0.37 ± 0.05 a	san218	0.22 ± 0.03 a
san45	0.73 ± 0.01 ab	san208	0.69 ± 0.03 a	san351	0.58 ± 0.02 ab	san568	0.32 ± 0.02 ab	san208	0.28 ± 0.03 ab
san351	0.75 ± 0.02 ab	san254	0.74 ± 0.02 ab	san254	0.58 ± 0.07 abc	aewolbudu	0.31 ± 0.05 abc	san568	0.23 ± 0.03 abc
san254	0.73 ± 0.01 bc	san568	0.73 ± 0.02 ab	san41	0.51 ± 0.09 abcd	san351	0.31 ± 0.01 abc	san187	0.22 ± 0.00 abc
san2daejogu	0.72 ± 0.07 bcd	san177	0.60 ± 0.04 abc	san135	0.52 ± 0.05 abcd	san187	0.30 ± 0.02 abc	aewolbudu	0.22 ± 0.03 bc
san184	0.73 ± 0.03 bcd	san2daejogu	0.65 ± 0.03 bcd	san187	0.50 ± 0.01 bcde	san180	0.25 ± 0.04 bcd	san184	0.21 ± 0.02 cd
san208	0.70 ± 0.03 cde	san45	0.77 ± 0.02 cde	san45	0.49 ± 0.05 bcdef	san41	0.27 ± 0.09 bcde	san180	0.20 ± 0.01 cde
san398	0.68 ± 0.06 def	san128	0.64 ± 0.06 cde	aewolbudu	0.47 ± 0.07 cdefg	san218	0.24 ± 0.02 bcdef	san135	0.19 ± 0.01 de
aewolbudu	0.66 ± 0.04 ef	san303	0.64 ± 0.08 cdef	san568	0.45 ± 0.02 defgh	san254	0.24 ± 0.04 cdef	san351	0.18 ± 0.01 ef
san41	0.67 ± 0.05 ef	san398	0.59 ± 0.07 def	san184	0.45 ± 0.08 defgh	san398	0.21 ± 0.04 defg	san86	0.17 ± 0.02 ef
san303	0.66 ± 0.05 ef	san184	0.59 ± 0.09 def	san180	0.43 ± 0.05 defgh	san2daejogu	0.21 ± 0.02 defg	suncheon	0.18 ± 0.01 ef
san128	0.64 ± 0.03 f.	san180	0.59 ± 0.08 def	san303	0.42 ± 0.14 defghi	suncheon	0.22 ± 0.04 defg	san41	0.15 ± 0.07 fg
suncheon	0.65 ± 0.04 f.	san135	0.59 ± 0.03 def	san177	0.40 ± 0.10 efghi	san135	0.20 ± 0.02 defg	san398	0.15 ± 0.03 fg
san177	0.63 ± 0.04 fg	aewolbudu	0.57 ± 0.03 ef	san128	0.40 ± 0.13 efghi	san45	0.20 ± 0.02 efgh	san2daejogu	0.15 ± 0.01 fg
san135	0.58 ± 0.03 gh	san41	0.62 ± 0.04 ef	san2daejogu	0.40 ± 0.04 fghi	san303	0.20 ± 0.08 fgh	san254	0.14 ± 0.01 gh
san218	0.52 ± 0.01 gh	suncheon	0.57 ± 0.06 ef	san218	0.37 ± 0.04 ghij	san86	0.19 ± 0.02 fgh	san45	0.13 ± 0.01 ghi
san180	0.57 ± 0.04 h	san187	0.56 ± 0.04 fg	suncheon	0.35 ± 0.06 hij	san184	0.19 ± 0.04 fgh	san9dangugdae	0.12 ± 0.00 hij
san86	0.49 ± 0.01 hi	san86	0.42 ± 0.02 gh	san398	0.31 ± 0.07 ij	san177	0.16 ± 0.03 gh	san303	0.10 ± 0.03 ij
san187	0.52 ± 0.03 hi	san218	0.42 ± 0.05 gh	san86	0.26 ± 0.04 j	san128	0.13 ± 0.04 h	san177	0.09 ± 0.01 j
san9dangugdae	0.45 ± 0.04 i	san9dangugdae	0.36 ± 0.06 h	san9dangugdae	0.22 ± 0.03 j	san9dangugdae	0.14 ± 0.01 h	san128	0.08 ± 0.02 j

*Means of $\pm$ standard deviation followed by different letters within columns are significantly different by Dunn test with Benjamini–Hochberg (BH) adjustment. Non-parametric rank data were used for statistical analysis; however, untransformed data are presented.

We were able to confirm the changes and trends of each germplasm over a five-month period that can be found by comparing Table 4 and Fig. 13. We observed that the differences in GCP rates between each germplasm were generally similar, with a few exceptions. In particular, the differences were maintained at 95% or higher in August and September. However, in contrast to the previous months, when san180 and san568 germplasms showed a GCP of 100%, they showed GCP of 67.22% and 60.97%, respectively, in October, a sharp decrease compared to September. On the other hand, san303 germplasm showed a GCP of 96.90% in August, ranking low, but it increased to 98.37% in October. Similarly, although the GCP of san187 germplasm decreased from 95.99% in August to 91.11% in September, it increased again to 94.87% in October. Based on the fact that the GCP of san180 and san568 germplasms decreased while those of san303 and san187 germplasms increased during the same period, we were able to observe that the changes in GCP vary depending on the germplasm over time. Looking at the GCP in November, the germplasm with the highest GCP was san208, which showed a GCP of 94%. San208 continuously showed a high GCP of 97.48% in August, 97.81% in September, and 96.86% in October, and although it slightly decreased in November, the rate of decrease was less than that of other germplasms.

Upon examining the RGB and GCP images for Suncheon, san218, and san187 in December we noticed that the soil part (sand) was identified as green. However, we believe that during the process of lightly washing away the sand dust on the grass that covers the moss before taking pictures, the moss was exposed again on the sand surface. To solve this distortion, we applied various VIs such as NDRE, SAVI, and EVI (Figs. 15, 16, 17, Tables 6, 7, 8). The results of NDRE show that san135, san180, san187, and san218 represent high values. On the other hand, the values of SAVI represent that san128 and san398 are higher values than others. Lastly, EVI applied results suggested that san128 got a high value. The average result of NDRE for san208 from August to December was found to be about 3% 5% respectively (Table 6). In constrast, SAVI decreased from August (38%) to December (4%) (Table 7). Furthermore, EVI remained slightly decreased during this time period (Table 8).

**Table 6:** Average differences of NDRE among germplasm for August to December.

Aug		Sep		Oct		Nov		Dec	
Germplasm	Measured value	Germplasm	Measured value	Germplasm	Measured value	Germplasm	Measured value	Germplasm	Measured value
san218	0.12 ± 0.01 a	san568	0.10 ± 0.01 a	san218	0.20 ± 0.02 a	san135	0.19 ± 0.03 a	san180	0.23 ± 0.01 a
san180	0.10 ± 0.01 ab	san398	0.09 ± 0.01 a	san187	0.17 ± 0.01 ab	san180	0.17 ± 0.02 a	san218	0.22 ± 0.01 a
san135	0.10 ± 0.01 ab	suncheon	0.08 ± 0.01 ab	san180	0.15 ± 0.02 abc	san218	0.16 ± 0.01 a	san135	0.2 ± 0.02 ab
san187	0.08 ± 0.01 bc	san86	0.07 ± 0.01 ab	san184	0.12 ± 0.02 bcd	san187	0.13 ± 0.01 ab	san187	0.19 ± 0.01 ab
san184	0.06 ± 0.01 cd	san180	0.07 ± 0.01 ab	san135	0.11 ± 0.01 cd	san184	0.09 ± 0.01 bc	san184	0.12 ± 0.01 bc
suncheon	0.05 ± 0.01 de	san177	0.06 ± 0.01 bc	san568	0.11 ± 0.02 cd	san86	0.08 ± 0.01 bc	san9dangugdae	0.11 ± 0.01 bcd
san398	0.05 ± 0.01 ef	san187	0.05 ± 0.01 c	san398	0.09 ± 0.01 de	suncheon	0.07 ± 0.01 cd	san568	0.10 ± 0.01 cd
san86	0.04 ± 0.01 f.	san208	0.05 ± 0.01 c	san9dangugdae	0.08 ± 0.01 ef	san568	0.07 ± 0.01 cde	suncheon	0.09 ± 0.01 cde
san303	0.04 ± 0.01 g	san9dangugdae	0.05 ± 0.01 c	suncheon	0.06 ± 0.01 fg	san398	0.06 ± 0.01 cdef	san86	0.09 ± 0.01 def
san177	0.04 ± 0.01 g	san128	0.05 ± 0.01 cd	san86	0.06 ± 0.01 fg	san9dangugdae	0.06 ± 0.01 defg	san45	0.06 ± 0.01 ef
san568	0.04 ± 0.01 g	san218	0.05 ± 0.01 de	san2daejogu	0.06 ± 0.01 fg	san41	0.05 ± 0.02 efgh	aewolbudu	0.06 ± 0.01 fg
san45	0.04 ± 0.01 g	san184	0.04 ± 0.01 def	san128	0.05 ± 0.01 gh	san2daejogu	0.05 ± 0.01 fghi	san177	0.05 ± 0.01 gh
san9dangugdae	0.03 ± 0.01 h	san2daejogu	0.04 ± 0.01 ef	san177	0.04 ± 0.01 hi	san351	0.04 ± 0.03 ghij	san398	0.05 ± 0.01 hi
san208	0.03 ± 0.01 h	aewolbudu	0.04 ± 0.01 ef	san208	0.04 ± 0.01 hij	san128	0.04 ± 0.01 hijk	san254	0.05 ± 0.01 hi
san128	0.03 ± 0.01 hi	san254	0.04 ± 0.01 ef	aewolbudu	0.04 ± 0.01 ij	san208	0.04 ± 0.01 hijk	san2daejogu	0.05 ± 0.01 hi
san254	0.03 ± 0.01 hij	san303	0.04 ± 0.01 ef	san254	0.03 ± 0.01 ij	aewolbudu	0.04 ± 0.01 ijk	san303	0.05 ± 0.01 hi
san2daejogu	0.03 ± 0.01 ij	san45	0.04 ± 0.01 fg	san45	0.03 ± 0.01 jk	san254	0.03 ± 0.01 jk	san208	0.05 ± 0.01 hij
aewolbudu	0.03 ± 0.01 j	san135	0.03 ± 0.01 gh	san41	0.03 ± 0.01 jk	san45	0.03 ± 0.01 jkl	san41	0.05 ± 0.01 hij
san41	0.02 ± 0.01 k	san41	0.02 ± 0.01 h	san351	0.02 ± 0.02 jk	san177	0.03 ± 0.01 kl	san128	0.05 ± 0.01 ij
san351	0.02 ± 0.01 k	san351	0.01 ± 0.01 h	san303	0.02 ± 0.01 k	san303	0.01 ± 0.01 l	san351	0.04 ± 0.01 j

*Means of $\pm$ standard deviation followed by different letters within columns are significantly different by Dunn test with Benjamini–Hochberg (BH) adjustment. Non-parametric rank data were used for statistical analysis; however, untransformed data are presented.

**Table 7:** Average differences of SAVI among germplasm for August to December.

Aug		Sep		Oct		Nov		Dec	
Germplasm	Measured value	Germplasm	Measured value	Germplasm	Measured value	Germplasm	Measured value	Germplasm	Measured value
san303	0.47 ± 0.02 a	san2daejogu	0.45 ± 0.02 a	san128	0.4 ± 0.02 a	san398	0.26 ± 0.03 a	san398	0.25 ± 0.03 a
san128	0.45 ± 0.01 a	san398	0.45 ± 0.01 a	san398	0.39 ± 0.03 a	san128	0.25 ± 0.02 a	san128	0.19 ± 0.02 ab
san177	0.44 ± 0.01 ab	san45	0.39 ± 0.01 ab	san177	0.35 ± 0.03 ab	san177	0.14 ± 0.02 ab	san218	0.12 ± 0.01 abc
san398	0.42 ± 0.02 bc	san177	0.28 ± 0.03 bc	san303	0.32 ± 0.02 abc	san303	0.13 ± 0.01 abc	san184	0.11 ± 0.01 bcd
san45	0.4 ± 0.02 cd	san184	0.26 ± 0.03 bc	san254	0.25 ± 0.04 bcd	san568	0.11 ± 0.02 bcd	san187	0.1 ± 0.01 cde
san254	0.38 ± 0.02 cd	san180	0.25 ± 0.02 cd	san45	0.24 ± 0.05 bcd	san45	0.11 ± 0.04 cd	san303	0.08 ± 0.01 de
san208	0.38 ± 0.01 de	san86	0.23 ± 0.01 cd	san208	0.23 ± 0.04 cd	san254	0.09 ± 0.02 de	san177	0.08 ± 0.01 ef
san184	0.33 ± 0.02 ef	san303	0.23 ± 0.01 cd	san86	0.18 ± 0.01 de	suncheon	0.09 ± 0.02 de	san135	0.08 ± 0.01 ef
san351	0.33 ± 0.02 f.	san41	0.23 ± 0.04 cd	san2daejogu	0.16 ± 0.03 ef	san86	0.09 ± 0.01 de	san568	0.07 ± 0.01 f.
san2daejogu	0.29 ± 0.04 g	san218	0.22 ± 0.02 cd	san568	0.16 ± 0.02 ef	san9dangugdae	0.09 ± 0.01 de	san180	0.07 ± 0.01 f.
aewolbudu	0.26 ± 0.01 gh	san128	0.21 ± 0.02 de	san184	0.14 ± 0.02 efg	san184	0.09 ± 0.02 de	san2daejogu	0.05 ± 0.01 g
san218	0.26 ± 0.01 gh	san187	0.19 ± 0.02 ef	aewolbudu	0.14 ± 0.02 fg	san2daejogu	0.07 ± 0.01 ef	san254	0.05 ± 0.01 g
san86	0.25 ± 0.02 ghi	san568	0.18 ± 0.03 ef	suncheon	0.14 ± 0.02 fg	san208	0.07 ± 0.02 fg	san45	0.04 ± 0.01 gh
san187	0.25 ± 0.01 hi	suncheon	0.18 ± 0.01 ef	san180	0.13 ± 0.02 fgh	aewolbudu	0.06 ± 0.01 fg	san208	0.04 ± 0.01 ghi
san180	0.23 ± 0.02 ij	aewolbudu	0.16 ± 0.02 fg	san41	0.12 ± 0.01 gh	san180	0.05 ± 0.01 fgh	aewolbudu	0.04 ± 0.01 hij
suncheon	0.21 ± 0.02 jk	san351	0.15 ± 0.02 fg	san9dangugdae	0.11 ± 0.01 hi	san41	0.05 ± 0.01 fgh	suncheon	0.04 ± 0.01 hij
san41	0.2 ± 0.02 jk	san254	0.14 ± 0.01 fg	san218	0.09 ± 0.02 i	san187	0.05 ± 0.01 gh	san9dangugdae	0.03 ± 0.01 ij
san568	0.18 ± 0.03 kl	san9dangugdae	0.12 ± 0.01 gh	san351	0.09 ± 0.02 i	san218	0.05 ± 0.01 gh	san351	0.03 ± 0.01 ij
san9dangugdae	0.14 ± 0.01 lm	san208	0.12 ± 0.02 gh	san187	0.08 ± 0.02 i	san351	0.05 ± 0.01 gh	san41	0.03 ± 0.01 j
san135	0.13 ± 0.01 m	san135	0.1 ± 0.01 h	san135	0.07 ± 0.01 i	san135	0.04 ± 0.01 h	san86	0.03 ± 0.01 j

*Means of $\pm$ standard deviation followed by different letters within columns are significantly different by Dunn test with Benjamini–Hochberg (BH) adjustment. Non-parametric rank data were used for statistical analysis; however, untransformed data are presented.

**Table 8:** Average differences of EVI among germplasm for August to December.

Aug		Sep		Oct		Nov		Dec	
Germplasm	Measured value	Germplasm	Measured value	Germplasm	Measured value	Germplasm	Measured value	Germplasm	Measured value
san128	0.23 ± 0.02 a	san128	0.19 ± 0.02 a	san135	0.05 ± 0.02 a	san128	0.06 ± 0.02 a	san128	0.05 ± 0.01 a
san45	0.17 ± 0.02 ab	san303	0.15 ± 0.03 ab	san184	0.04 ± 0.02 a	san184	0.01 ± 0.01 ab	san184	0.01 ± 0.01 ab
san254	0.12 ± 0.02 abc	san254	0.13 ± 0.02 ab	san254	0.03 ± 0.02 ab	san45	0.01 ± 0.01 abc	san351	0.01 ± 0.01 abc
san184	0.11 ± 0.02 abc	san45	0.13 ± 0.03 ab	san2daejogu	0.02 ± 0.01 abc	san351	0.01 ± 0.01 abcd	san2daejogu	0.01 ± 0.01 abcd
san351	0.1 ± 0.02 abc	san177	0.11 ± 0.04 ab	san128	0.02 ± 0.02 abc	san41	0.01 ± 0.01 abcd	san41	0.01 ± 0.01 abcd
san2daejogu	0.09 ± 0.03 abc	san208	0.09 ± 0.05 abc	san208	0.02 ± 0.02 abc	san2daejogu	0.01 ± 0.01 bcd	san398	0.01 ± 0.01 bcde
san303	0.09 ± 0.05 bcd	san184	0.04 ± 0.02 bcd	san351	0.02 ± 0.01 abc	san254	0.01 ± 0.01 cd	san254	0.01 ± 0.01 cdef
san208	0.06 ± 0.04 cde	san351	0.02 ± 0.01 cd	san45	0.02 ± 0.01 abc	san135	0.01 ± 0.01 cd	san45	0.01 ± 0.01 defg
san41	0.05 ± 0.02 cdef	san2daejogu	0.02 ± 0.02 de	san180	0.01 ± 0.01 bcd	san180	0.01 ± 0.01 de	san187	0.01 ± 0.01 defg
san398	0.03 ± 0.03 defg	san180	0.01 ± 0.01 def	san41	0.01 ± 0.01 cde	san398	0.01 ± 0.01 ef	san135	0.01 ± 0.01 efgh
san177	0.02 ± 0.02 defg	san41	0.01 ± 0.01 def	suncheon	0.01 ± 0.01 def	san208	0.01 ± 0.01 fg	san180	0.01 ± 0.01 fghi
san218	0.01 ± 0.01 efg	san187	0.01 ± 0.01 efg	san86	0.01 ± 0.01 def	aewolbudu	0.01 ± 0.01 fg	san208	0.01 ± 0.01 fghi
aewolbudu	0.01 ± 0.01 fg	san86	0.01 ± 0.01 fgh	san187	0.01 ± 0.01 def	suncheon	0.01 ± 0.01 fgh	aewolbudu	0.01 ± 0.01 fghi
suncheon	0.01 ± 0.01 g	san135	0.01 ± 0.01 ghi	aewolbudu	0.01 ± 0.01 ef	san9dangugdae	0.01 ± 0.01 fghi	san218	0.01 ± 0.01 ghij
san187	0.01 ± 0.01 g	suncheon	0.01 ± 0.01 ghi	san303	0.01 ± 0.01 ef	san187	0.01 ± 0.01 fghi	san568	0.01 ± 0.01 hij
san180	0.01 ± 0.01 g	san9dangugdae	0.01 ± 0.01 ghi	san568	0.01 ± 0.01 ef	san177	0.01 ± 0.01 fghi	san303	0.01 ± 0.01 hijk
san86	0.01 ± 0.01 g	aewolbudu	0.01 ± 0.01 ghi	san9dangugdae	0.01 ± 0.01 f.	san86	0.01 ± 0.01 ghi	san177	0.01 ± 0.01 ijk
san135	0.01 ± 0.01 g	san218	0.01 ± 0.01 hi	san177	0.01 ± 0.01 f.	san568	0.01 ± 0.01 hi	san9dangugdae	0.01 ± 0.01 ijk
san9dangugdae	0.01 ± 0.01 g	san398	0.01 ± 0.01 i	san398	0.01 ± 0.01 f.	san218	0.01 ± 0.01 i	suncheon	0.01 ± 0.01 jk
san568	0.01 ± 0.01 g	san568	0.01 ± 0.01 i	san218	0.01 ± 0.01 f.	san303	0.01 ± 0.01 i	san86	0.01 ± 0.01 k

*Means of $\pm$ standard deviation followed by different letters within columns are significantly different by Dunn test with Benjamini-Hochberg (BH) adjustment. Non-parametric rank data were used for statistical analysis; however, untransformed data are presented. Because the size of the EVI values is so small that some of the values are rounded to two decimal places.

The differences and patterns of changes in NDVI for each genetic resource, provided a comprehensive understanding of the changes in NDVI over the 5-month period (Table 5 and Fig. 14). In August, the san568 genetic resource showed the highest NDVI value of 0.85, while the san9dangugdae genetic resource showed the lowest NDVI value of 0.45. Checking the NDVI values in October, it can be observed that the changes in NDVI values vary greatly depending on the genetic resource, similar to the GCP. The NDVI value of san568 decreased to 0.45, while the san208 genetic resource showed the best value of 0.65. The san9dangugdae genetic resource consistently showed the lowest NDVI value, which continued until November. In December, the highest NDVI value was 0.32 for the san218 genetic resource, while the lowest NDVI value was 0.08 for the san128 genetic resource.

**Figure 18:**
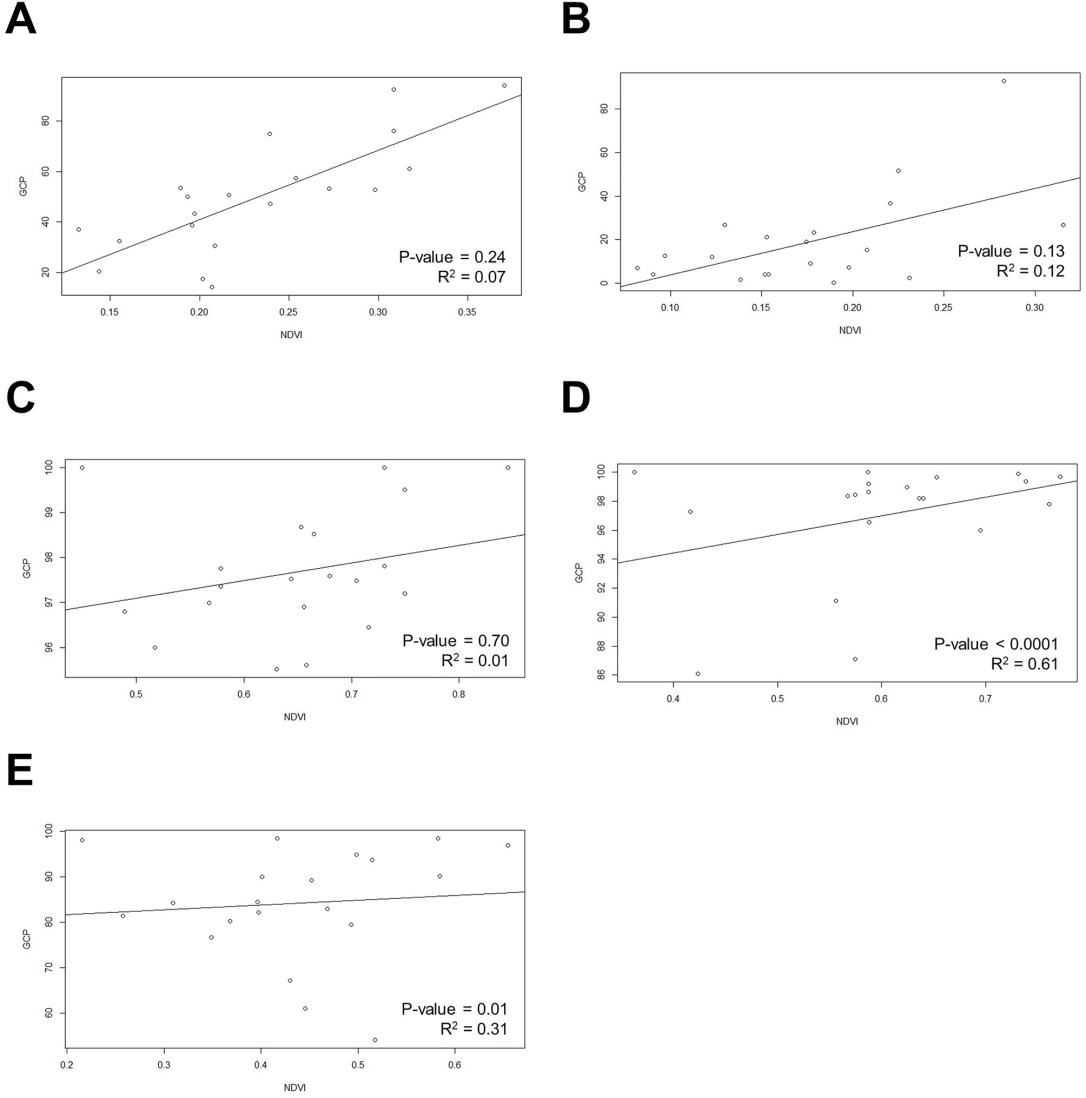
RGB, GCP and NDVI image of twenty turf germplasms.

Based on the analysis of the correlation between the growth rate and NDVI for each month (Fig. 18), November and December showed a significant correlation with a *p*-value less than 0.05 and an r square-values of 0.61 and 0.31.

## Discussion

Turfgrass is a valuable ground cover globally, providing numerous environmental, economic, and social benefits. To assess overall plant health and establishment rate over time, green cover percent, which measures the percentage of green vegetation per unit area, is a crucial indicator. Turfgrass producers and consumers prefer species and cultivars with faster establishment from plugs or sprigs and higher GCP. The conventional method of visually assessing turfgrass plots to estimate the amount of green cover, either on a percent or 1–9 scale, has become a standard and is regularly employed by turfgrass researchers. Low temperatures can have a detrimental effect on grass, but certain grasses exhibit higher resistance due to increased enzyme activity, unsaturated fatty acid content, and anthocyanin production^18^.

**Figure 19:**
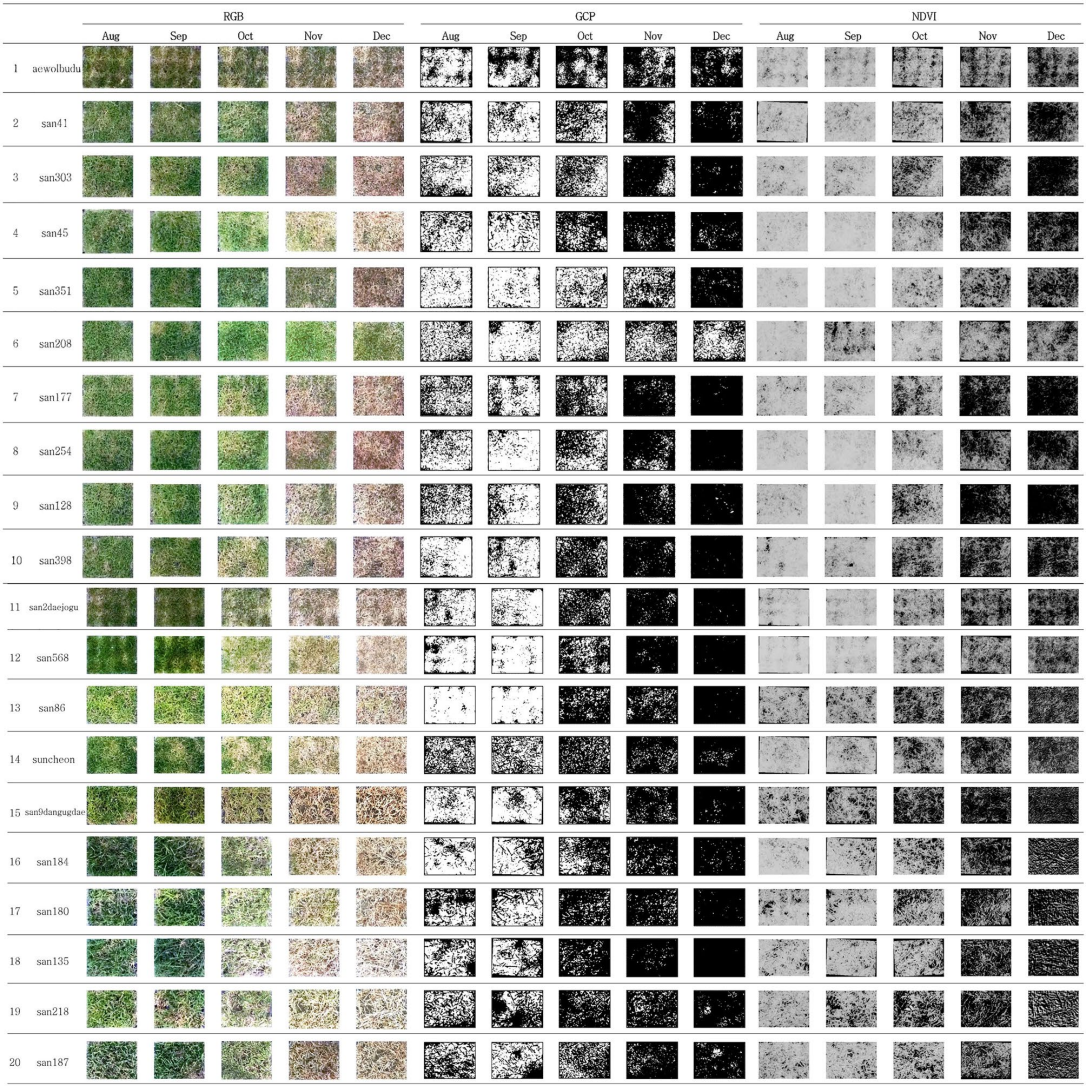
Pearson correlation coefficient of GCP and NDVI per month. (**A**) August, (**B**) September, (**C**) October (**D**) November (**E**) December.

In this study, we aim to objectively evaluate the quality of grass by proposing criteria for its assessment using an RGB camera and a Multi-spectral sensor. The amount and quality of green in grass per unit area and the biomass of the plant have been shown to be correlated with its cold tolerance^37^ Furthermore, color is a component of visual evaluation for grass quality^38^. Through analysis of RGB images, it is possible to perform evaluations similar to those made by the human eye, and in particular, to determine the proportion of green area by counting the number of pixels, allowing us to assess the amount of green in the grass. The Normalized Difference Vegetation Index (NDVI) is a widely used index for the quantitative estimation of vegetation, which uses the reflectance of near-infrared and red wavelengths to quantify plant growth^39^. Moreover, NDVI is the most commonly used index for greenness and can estimate the amount of chlorophyll that makes plants appear green by using reflectance, which allows us to assess the quality of grass greenness using NDVI in this study^40^. All genetic resources showed a GCP of over 95% in August. However, looking at the changes until October, it was found that some genetic resources (san303 and san187) showed a slight increase in greenness rate, indicating that these two genetic resources continued to grow relatively longer compared to other genetic resources, given that the growing period of Korean grass is from April to mid-October. However, most of the genetic resources showed a decrease in the greenness rate in October. This was more pronounced in the final result in December, where the san208 genetic resource maintained a very excellent greenness rate of over 90%, while the san125 genetic resource showed a greenness rate of 0.4%, showing a very clear difference from san208. These results indicate that traits related to changes in greenness rate are expressed differently in each genetic resource. During the research conducted from August to December 2021 at a grass cultivation site, the graph showing the highest temperature, lowest temperature, and average temperature obtained from the nearby weather observatory (Fig. 2) shows a tendency for the temperature to decrease as time passes from August to December, especially showing a rapid decrease in temperature in mid-October. By looking at the temperature graph (Fig. 2) and Fig. 19 together, it can be seen that in August and September, all grass genetic resources showed good quality green color at appropriate temperatures for growth, but in mid-October, the temperature decreased rapidly and the color of the grass changed from green to yellow or yellow–brown in most genetic resources. Low-temperature stress is one of the most harmful environmental stress factors for grass, leading to decreased photosynthesis due to photoinhibition, accumulation of carbohydrates within the cell, dehydration due to cell membrane damage caused by ice crystals formed inside and outside the cell at temperatures below freezing, changes in cell membrane composition, and protein denaturation, which can cause the grass to wither and die in severe cases^41^. Grass that is resistant to low temperatures has higher levels of the enzyme activity associated with carbon metabolism, the unsaturated fatty acid content in the lipids that make up the cell membrane, and accumulation of proline, which maintains membrane stability and scavenges reactive oxygen species, than susceptible grasses^5^. In addition, grasses produce and accumulate anthocyanin, an antioxidant, as a secondary metabolite in response to low-temperature stress, which reduces oxidative damage and gives winter grass a red color visible to the naked eye^18^ In our study, we found that In November and December, the grass color changed noticeably to not only yellow but also red and reddish-blue. The change in grass color to red is due to the synthesis, transportation, and accumulation of anthocyanin, a secondary metabolite, and antioxidant known to prevent oxidative damage to grass caused by the large number of reactive oxygen species generated inside the plant under low temperatures. A study shows that the accumulation of anthocyanin is related to the high cold tolerance of grass^18^.

Bremer et al.'s^42^ studied, the quality of grass was evaluated by calculating the NDVI from canopy images of grass obtained through a hyperspectral sensor, which showed a high correlation (R2 = 0.88, *p* < 0.0001) with the visually evaluated green cover area of the grass. They also mentioned that the RED image, which is part of the NDVI, affects the density difference and chlorophyll content of grass, and that additional research is needed, particularly using the NIR image, to predict the water stress status of plants before stress symptoms appear. In this study, considering the growth period of Korean grass, during the growing season in August and September, all grasses were observed to have high NDVI values as they appeared green. However, from October to December, during the non-growth season when the grass entered dormancy and lost its green color, the NDVI values gradually decreased. Among the genetic resources studied, san218 showed the highest NDVI value in December. In September, it showed a very low NDVI value of 0.42, but in December, it showed a higher NDVI value of 0.32 compared to other genetic resources. In contrast, san9dangugdae showed a higher NDVI value of 0.45 in August compared to san218, but a relatively low NDVI value of 0.12 in December among the genetic resources. Like the rate of greening, the rate of change in NDVI values also showed a significant difference in the change patterns of genetic resources. However, while the NDVI values of other genetic resources decreased as the greening rate decreased, only san208 showed a high greening rate of over 90% from August to December, but the NDVI value gradually decreased over time. This seems to be because san208 showed an increase in the number of other pigments produced under low-temperature stress, reducing the relative proportion of chlorophyll and decreasing the NDVI value. Therefore, additional analysis of leaf pigment composition for san208 is needed to explain why it still showed high greening compared to other genetic resources.

Significant results were only obtained for November when examining the correlation between the greenness rate and NDVI. This indicates that there is almost no correlation between the trait of maintaining quality greenness by occupying a certain area with grass (greenness rate data confirmed by RGB camera) and the trait of maintaining quality greenness by the amount of chlorophyll in the leaves themselves (NDVI based on multi-spectral imagery). The correlation that appeared in November, when the growth status of the grass was extremely poor, is believed to have occurred because both the qualitative state (NDVI) and quantitative state (greenness rate) of the grass showed a correlation. Previously, even if the qualitative state of the NDVI value was low, there appeared to be many grass cultivars with high quantitative states that could not show a correlation. In December, even superior cultivars showed a decrease in both the qualitative and quantitative states, which is presumed to be the reason why the correlation did not appear again. Therefore, it is necessary to select the exact observation time to confirm the correlation between the traits obtained from the two images.

In a study conducted by Barboza et al.^43^, researchers used multiple linear regression analysis to determine the most accurate estimates for fresh biomass. They considered three input parameters: plant width, height, and vegetation index (either NDVI or NDRE). While NDRE showed saturation during the reproductive stages, the results of the analysis revealed that NDVI performed better than NDRE in estimating fresh biomass. This means that NDVI provided more reliable and accurate predictions of biomass compared to NDRE. Despite its limitations during the reproductive stages, NDVI was found to be a more suitable vegetation index for estimating fresh biomass in this particular study. In our study, we examined various vegetation indices, including NDRE, SAVI, and EVI. The results of NDRE indicated that san135, san180, san187, and san218 had high values. Conversely, the values of SAVI showed that san128 and san398 had higher values compared to others. Lastly, when EVI was applied, san128 had a high value. On average, the NDRE results for san208 from August to December ranged from approximately 3% to 5%. In contrast, SAVI decreased from August (38%) to December (4%). Furthermore, EVI showed a slight decrease during this time period. The variation may be due to the fact that the NDRE index is better suited for evaluating chlorophyll content in larger trees, while the NDVI index is more reliable for assessing biomass and health in smaller trees. The choice of index depends on the specific characteristics of the trees under study, such as their size and the desired parameter to be measured.

Boiarskii and Hasegawa conducted a study in 2019 where they compared two layers with different indices, specifically NDVI and NDRE, and observed variations in vegetation activity. The study found that NDVI proved to be particularly effective in analyzing large land areas to assess vegetation density and the overall greenness of crops. It provided insights into crop health, cultivation effectiveness, and seeding rates. On the other hand, NDRE allowed for the visualization of chlorophyll content in leaves. This suggests that different indices may be more suitable for different crops, plant densities, seeding rates, and growth stages^44^.

The response of plants to low-temperature stress can serve as indicators for research on cold tolerance. One method is to analyze digital images using RGB sensors to observe grass color and growth, while multispectral sensors can be used to evaluate grass quality in terms of color, density, and vegetation vitality. Multispectral imaging can provide information on chlorophyll content, canopy structure, and leaf area index at different wavelengths, and vegetation indices derived from them can be combined to assess plant moisture and nutrient status under environmental stress^45–47^. Fluorescence sensors can also be used to measure leaf chlorophyll fluorescence to evaluate the photosynthetic apparatus' state under low-temperature stress. A laser-induced chlorophyll fluorescence measurement method has also been proposed for outdoor measurement where ambient light and temperature are not constant^48,49^. Moreover, LIDAR/laser sensors can generate 3D images that can be used to examine plant structure, biomass, and other factors, providing a way for real-time research on the morphological changes in grass that are exposed to low-temperature stress^50,51^.

## Conclusion

This study suggests that the choice between using RGB-based greenness and NDVI-based greenness for evaluating grass greenness may vary depending on the purpose. When evaluating greenness from an economic standpoint, it may be more practical to use RGB-based greenness, which provides an intuitive estimate of the amount of greenness similar to visual observation. However, to select grass that maintains a high level of greenness, using NDVI-based greenness, which evaluates the grass from a biological perspective, can complement the RGB-based greenness. Therefore, it is recommended to use both greenness measures in a complementary manner to select grass that can maintain good greenness over time. Additionally, considering the time when the two measures show a correlation can be advantageous in selecting grass using both measures. Finally, by using the greenness measures of this study, it is expected that time and energy spent on grass quality evaluation can be reduced while selecting excellent quality grass through an objective and reproducible method.
